# The first evidence for Late Pleistocene dogs in Italy

**DOI:** 10.1038/s41598-020-69940-w

**Published:** 2020-08-07

**Authors:** Francesco Boschin, Federico Bernardini, Elena Pilli, Stefania Vai, Clément Zanolli, Antonio Tagliacozzo, Rosario Fico, Mariaelena Fedi, Julien Corny, Diego Dreossi, Martina Lari, Alessandra Modi, Chiara Vergata, Claudio Tuniz, Adriana Moroni, Paolo Boscato, David Caramelli, Annamaria Ronchitelli

**Affiliations:** 1 U.R. Preistoria e Antropologia, Dipartimento di Scienze Fisiche, della Terra e dell’Ambiente, Università degli Studi di Siena, Via Laterina 8, 53100 Siena, Italy; 2 Centro Fermi-Museo Storico della Fisica e Centro di Studi e Ricerche Enrico Fermi, Piazza del Viminale 1, 00184 Rome, Italy; 3 Multidisciplinary Laboratory, The Abdus Salam International Centre for Theoretical Physics, Via Beirut 31, 34151 Trieste, Italy; 4 Laboratory of Anthropology -Molecular Anthropology and Forensic Unit, Department of Biology, University of Florence, Firenze, Italy; 5 Laboratoire PACEA, UMR 5199 CNRS, Université de Bordeaux, Bâtiment B8, allée Geoffroy Saint Hilaire, 33615 Pessac Cedex, France; 6 Bioarchaeology Section of Museo delle Civiltà, Museo Nazionale Preistorico Etnografico “Luigi Pigorini”, Piazza G. Marconi 14, 00144 Rome, Italy; 7 Centro di Referenza Nazionale per la Medicina Forense Veterinaria, Istituto Zooprofilattico Sperimentale delle Regioni Lazio e Toscana “M. Aleandri”, Viale Europa, 30, 58100 Grosseto, Italy; 8 INFN (Istituto Nazionale di Fisica Nucleare) Sezione di Firenze, Via Sansone 1, 50019 Sesto Fiorentino, FI Italy; 9 Département Homme & Environnement, Muséum National d’Histoire Naturelle, UMR 7194, CNRS, Musée de l’Homme, Paris, France; 10 Sincrotrone Trieste S.C.p.A., AREA Science Park, Basovizza, Trieste Italy; 11 Centre for Archaeological Science, University of Wollongong, Northfields Avenue, Wollongong, NSW 2522 Australia; 12 Centro Studi sul Quaternario Onlus, Sansepolcro, Arezzo, Italy; 13 Istituto Italiano di Paleontologia Umana, Roma, Italy

**Keywords:** Archaeology, Evolutionary genetics, Palaeoecology

## Abstract

The identification of the earliest dogs is challenging because of the absence and/or mosaic pattern of morphological diagnostic features in the initial phases of the domestication process. Furthermore, the natural occurrence of some of these characters in Late Pleistocene wolf populations and the time it took from the onset of traits related to domestication to their prevalence remain indefinite. For these reasons, the spatiotemporal context of the early domestication of dogs is hotly debated. Our combined molecular and morphological analyses of fossil canid remains from the sites of Grotta Paglicci and Grotta Romanelli, in southern Italy, attest of the presence of dogs at least 14,000 calibrated years before present. This unambiguously documents one of the earliest occurrence of domesticates in the Upper Palaeolithic of Europe and in the Mediterranean. The genetic affinity between the Palaeolithic dogs from southern Italy and contemporaneous ones found in Germany also suggest that these animals were an important common adjunct during the Late Glacial, when strong cultural diversification occurred between the Mediterranean world and European areas north of the Alps. Additionally, aDNA analyses indicate that this Upper Palaeolithic dog lineage from Italy may have contributed to the genetic diversity of living dogs.

## Introduction

Dogs were the first animals domesticated by humans, long before the advent of agriculture^1^. Besides occupying a special place in our present day lives, dogs had important functional and symbolic roles throughout human history. However, the spatiotemporal context of their early domestication is debated from both archaeological and genetic perspectives: there is scant consensus on the location of first domestication centres, and the presence of one or more domestication events^1–5^, as well as a debate on the correct identification of the oldest archaeological specimens considered to represent dogs^6–11^. Latest genetic models suggest the presence of dogs in Europe at least 15,000 years ago, and a divergence between dogs and wolves between about 20,000 and 40,000 years ago^5,12^. Earlier potential dog domestication attempts may be represented by canid remains from Northern and Eastern Europe, and Russia^6–9,13–15^, even if their attribution to dogs or wolves is debated^5,10,16–20^. The earliest archaeological specimens unequivocally attributed to dogs lived around 16,000 years ago^21–23^, and were related to Magdalenian contexts in Western Europe. Available genetic evidence suggests that the domestication process leading to the current diversity of dogs took place in Europe^12^, even if a possible second event of domestication may also have occurred in Eastern Asia^5,24^. We present here the first evidence for Late Pleistocene dogs from two Upper Palaeolithic sites in southern Italy: Grotta Paglicci (Apulia, Foggia) and Grotta Romanelli (Apulia, Lecce). This is the oldest evidence of dogs in the Mediterranean.

**Figure 1:**
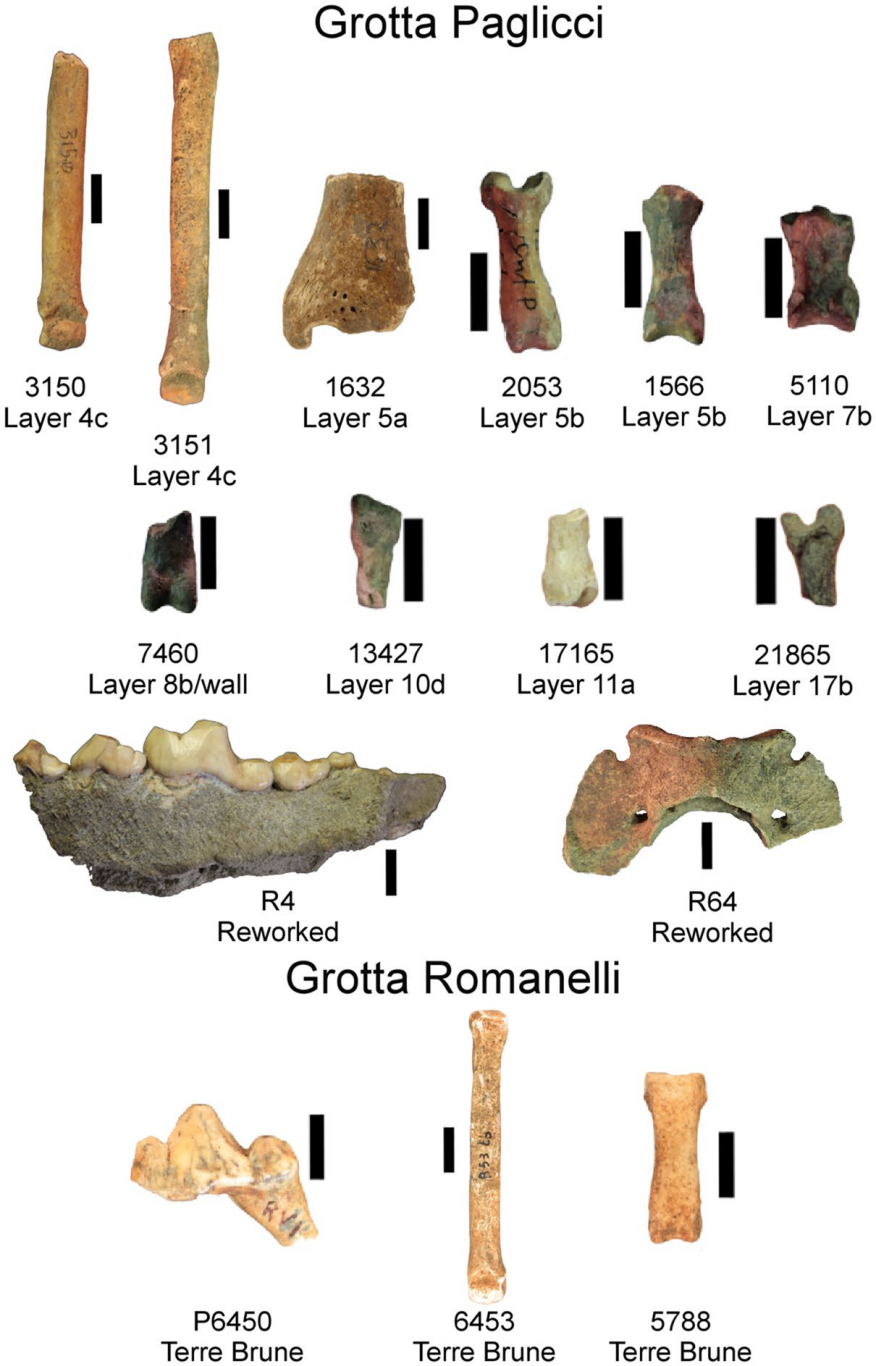
Pictures of the fifteen canid specimens from Grotta Paglicci and Grotta Romanelli. The post-cranial elements are in dorsal view (with the exception of 7460, ventral view), while the mandible R4 and the first lower molar P6450 are in buccal view. Scale bar, 1 cm.

Grotta Paglicci opens at about 143 m a.s.l. on the south-western slope of the Gargano promontory (Apulia, southern Italy) (Supplementary Figure 1). The present-day cave and a rock shelter of this site were originally part of a larger hypogean system. Researches at Paglicci have been carried out for over 50 years, first by the Museo Civico di Storia Naturale di Verona and, since 1971, by the University of Siena, in collaboration with the Soprintendenza Archeologia, Belle Arti e Paesaggio per le Province di Barletta – Andria – Trani e Foggia ^25^. The sediments in the rock shelter yielded Early Middle Palaeolithic and Acheulean stone tools^26,27^. A deep stratigraphic sequence is deposited in the cave, comprising Lower, Middle and Upper Palaeolithic^28–31^. The Upper Palaeolithic sequence uncovered inside the cave is one of the most complete in Europe and spans from the Aurignacian (about 39,000 years ago), notably characterized by the presence of marginally backed bladelets, to the Final Epigravettian (about 13,000 years ago)^30^. In addition to the large number of artifacts and faunal remains^31,32^, Grotta Paglicci yielded several human specimens^33^, as well as mobiliary symbolic objects (engraved stones and bones) and the only Upper Palaeolithic wall paintings discovered in Italy so far^34–37^. Among the faunal remains, here we analyse twelve *Canis* remains that show remarkably small dimensions or a reduced size of the lower first molar (3150, 3151, 1632, 1566, 2053, 5110, 7460, 13427, 17165, 21865, R4, R64; Fig. 1 and Supplementary Table 1). Almost all of them come from a secure stratigraphic context, with the exception of the mandible R4 and the atlas R64 that come from a reworked area of the deposit. Sieving of the reworked sediment from this area yielded materials exclusively related to the Evolved and Final Epigravettian. Direct ^14^C dating of the mandible R4 gave an age of 15,800–11,200 cal. yr bp (Supplementary Table 2). Another direct ^14^C date was obtained for the third metatarsal 3150 from the layer 4c (14,372–13,759 cal. yr bp).

Grotta Romanelli is located in Southern Apulia and opens at about 7 m a.s.l (Supplementary Figure 1). Cave stratigraphy is delimited at the bottom by a Tyrrhenian marine terrace (MIS 5) and consists of two main parts, the lowest of which is called “Terre rosse” and comprises all of the levels below the stalagmite F (dated to 40,000 ± 3,250 by ^230^Th/^238^U method) ^38–40^. The upper part of the deposit is called “Terre brune” (dated between about 13,800 and at least 8,600 cal. yr BP)^41^ and yielded Final Epigravettian artefacts together with a vertebrate fauna dominated by red deer, European ass and aurochs among ungulates and *Tetrax tetrax* among birds^42,43^. The three *Canis* remains discussed in this paper come from the “Terre brune” (6453, 5788 and P6450; Fig. 1 and Supplementary Table 1).

**Figure 2:**
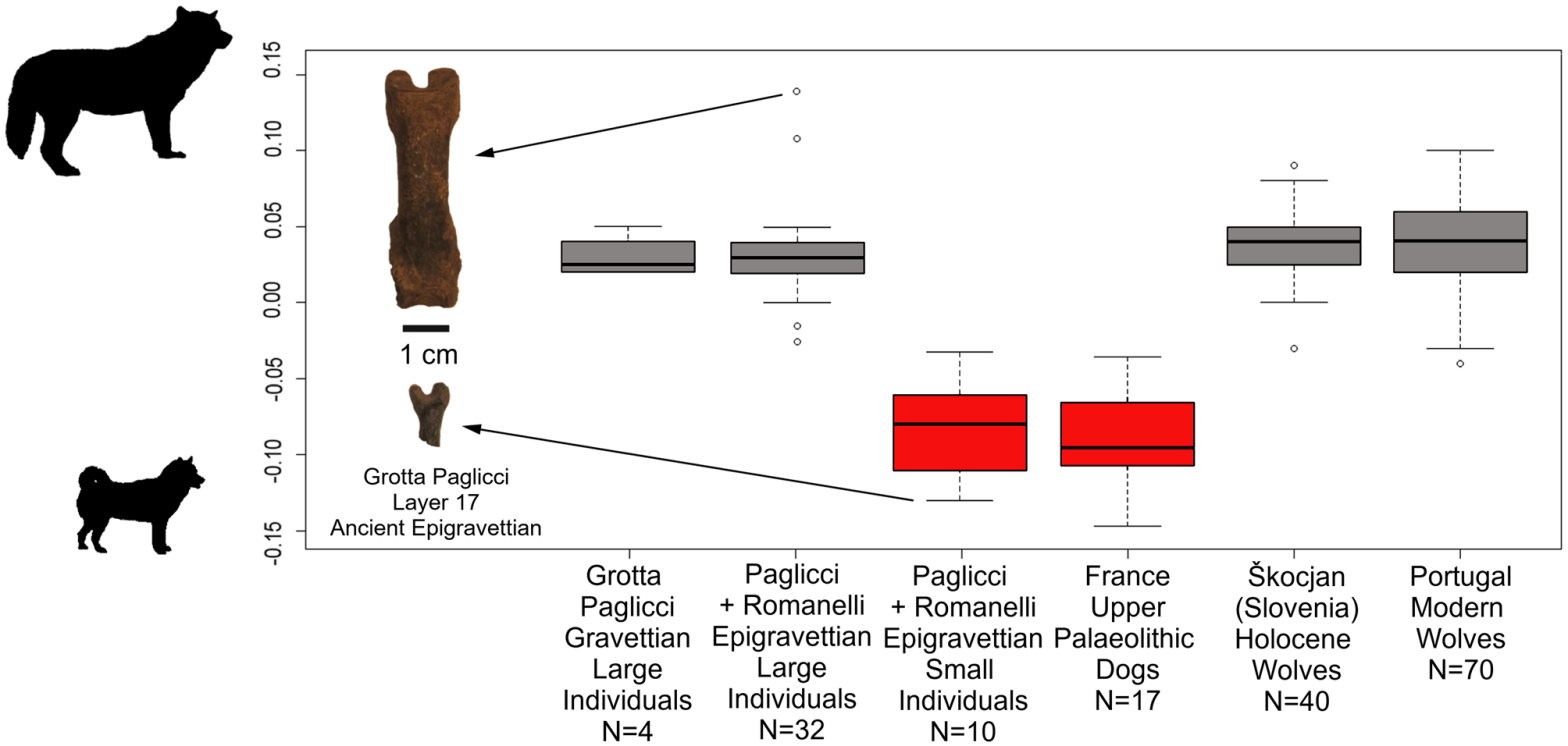
Log-ratio diagram of postcranial elements showing dimension of *Canis* remains compared with a standard (Supplementary Data S1). Negative values: specimens smaller than the standard; positive values: specimens larger than the standard. Silhouettes on the left indicate the difference in size between the largest individuals (wolves, grey plots) and the smallest ones (dogs, red plots). The two specimens illustrated in the box represent the two extremes of variation found at Grotta Paglicci (Epigravettian). It is worth noting that both of these first phalanges (the largest is 21930 and the smallest is 21865) come from the layer 17 (dated to about 20,000 cal. yr bp)^31^.

The fifteen dental and skeletal elements from Grotta Paglicci and Grotta Romanelli analysed here (Supplementary Table 1) represent small-bodied individuals or individuals with a lower first molar of reduced size. In addition to these canid remains, *Canis* specimens from the Upper Palaeolithic levels of these two Italian sites also include large individuals, similar in size to the extant European wolves, resulting in a remarkable dimensional variation (Fig. 2). We measured and compared the size of the post-cranial elements from both sites. Whenever the epiphyses of long bones were lacking or if the bones were burned, we applied an ad-hoc X-ray microtomography (μCT) protocol to evaluate the ontogeny of bone tissues^44^ or the heat-induced shrinkage^45^ (see "Methods" section). As a result, all long bones considered here were fully developed; among the burnt ones, only a first metacarpal (specimen 17165, Fig. 1) shows internal fractures compatible with shrinkage (Supplementary Figure 2). Wolves are predators characterized by fast body growth^46^, and they reach the minimum adult size when about one year old. This is a relevant characteristic as the smallest bones studied here show complete skeletal development, meaning that they represent small-bodied adult individuals and do not belong to still-growing individuals of larger size. We compared postcranial biometric variables measured on *Canis* remains from Grotta Paglicci, Grotta Romanelli, and from other Pleistocene to Holocene European sites, as well as to extant populations of wild individuals with a standard (a complete skeleton of a present-day female wolf from Italy, Supplementary Table 3) using a log-shape ratio method to estimate the relative body size of each population^47^. Our results show that the Gravettian specimens from Grotta Paglicci, as well as the Epigravettian larger individuals from Grotta Paglicci and Grotta Romanelli, were similar to those of other wild populations (extant wolves from Portugal and Holocene archaeological specimens from Slovenia), while the Epigravettian smaller individuals from Southern Italy (Grotta Paglicci and Romanelli) showed dimensions comparable to those of Palaeolithic dogs from France (Fig. 2).

In order to extract the maximum information from the teeth, we applied approaches that are commonly used in virtual paleoanthropology to assess the internal tooth structural signature^48,49^. The analysis of the tooth crown tissue proportions of P6450 from Grotta Romanelli was performed on a limited portion of the crown to avoid the influence of occlusal wear (see "Methods" section).

**Figure 3:**
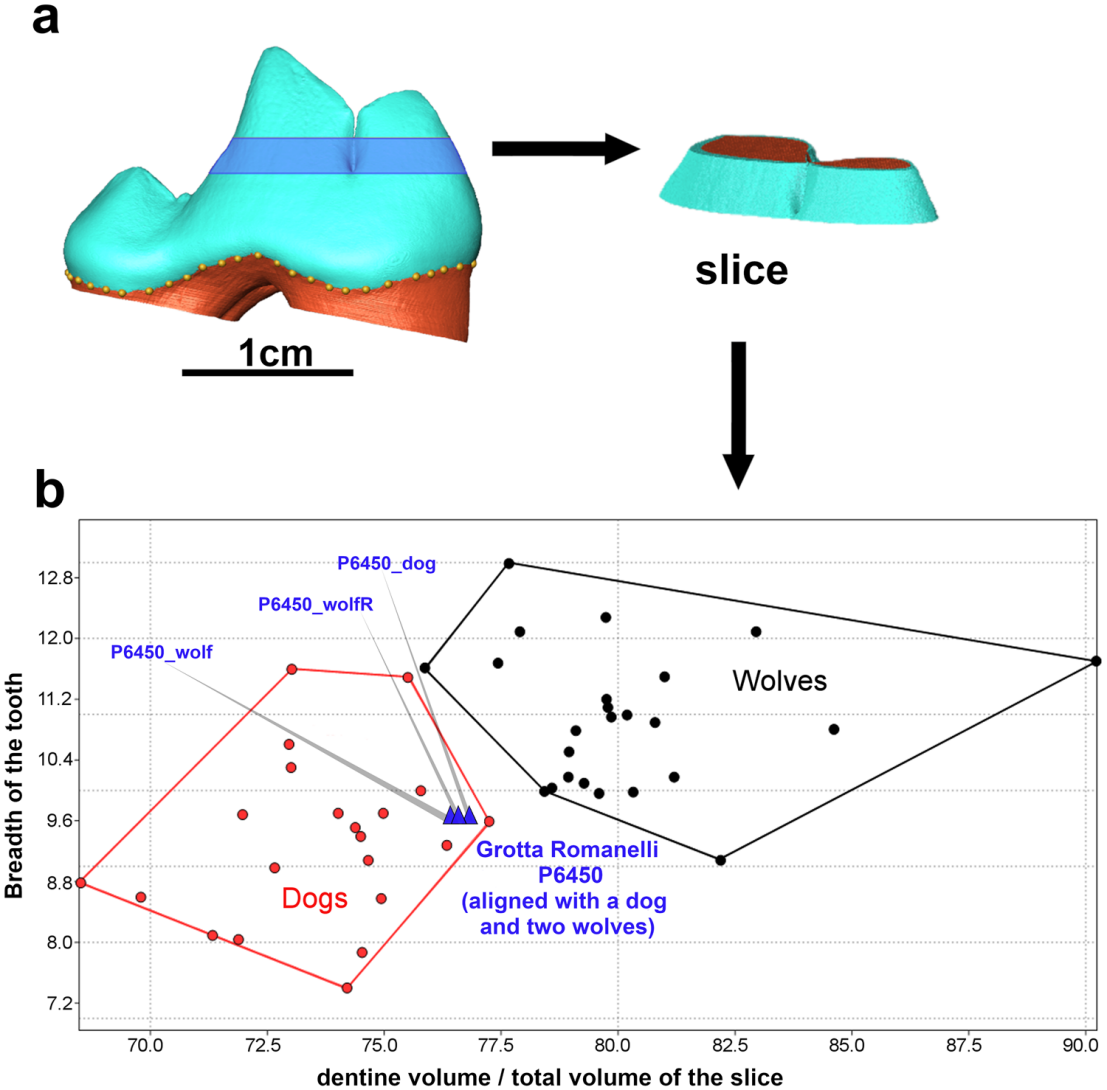
Tooth crown tissue proportions. (**a**) Virtual extraction of a tooth slice from the lower first molar. (**b**) Variability of the lower first molar percent of crown dentine (X-axis) and breadth of the tooth (Y-axis) in dogs and wolves. Tooth P6450 from Grotta Romanelli is represented by the three blue triangles (see "Methods" section and Supplementary Table 4).

Nevertheless, using this method focusing on the protocone-paracone region (Fig. 3a), we detected significant differences in crown dentine proportions between the 21 dog and 23 wolf individuals of our comparative sample (Supplementary Table 4 and Supplementary Figure 3). The percent of crown dentine is statistically higher in wolves than in dogs (Mann–Whitney *U* test *p*-value < 0.05), including for the smaller wolf individuals showing a tooth size comparable to that of larger dogs. Our estimates for the specimen P6450 show low percent of crown dentine, falling closer to the average dog value than to the higher mean value of wolves (Fig. 3b).

**Figure 4:**
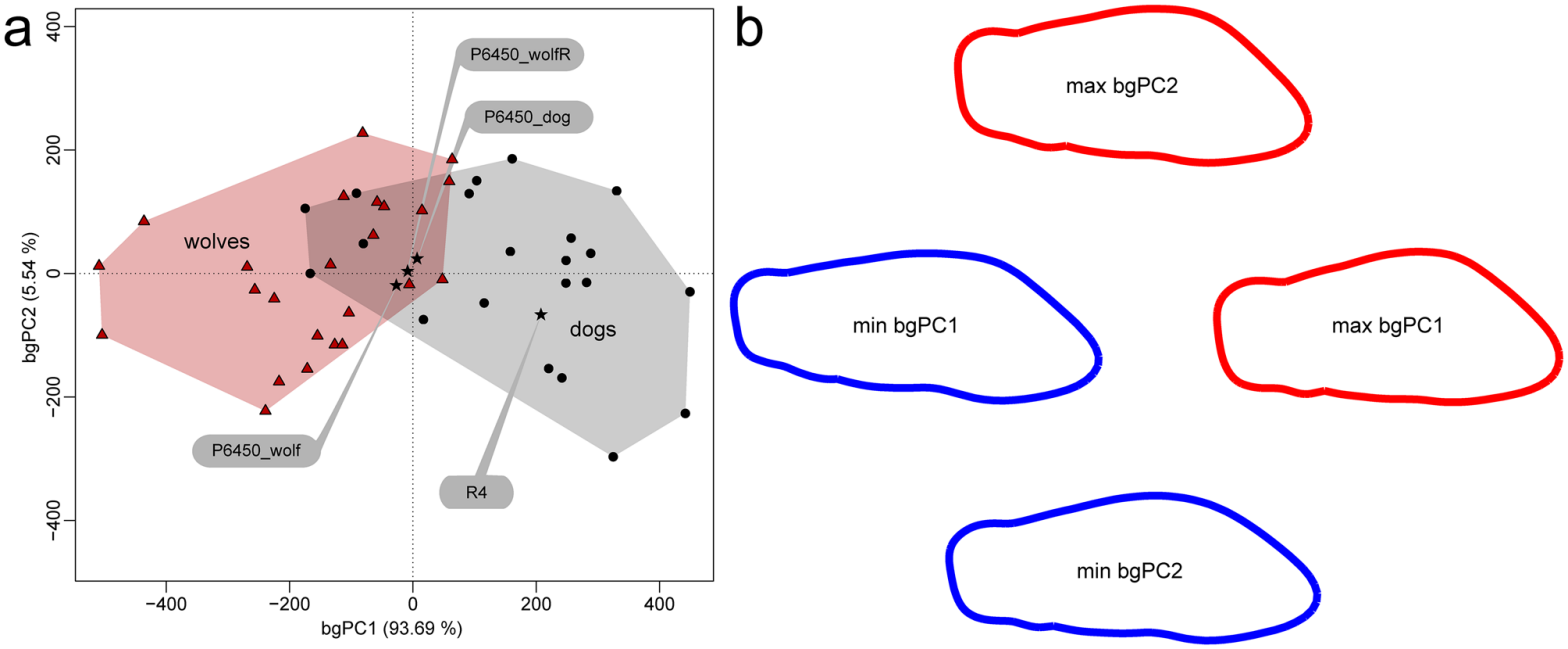
Geometric morphometric analyses of the lower first molar crown outline. (**a**) Between-group principal component analyses of the 2D landmark Procrustes-registered shape coordinates of the lower first molar outline of R4 and P6450 compared with dogs and wolves. (**b**) Extreme shapes along bgPC1 and bgPC2 (see "Methods" section and Supplementary Table 10).

We also performed geometric morphometric analyses of the crown occlusal outline of the lower carnassial tooth of the mandible R4 from Grotta Paglicci and of P6450 from Grotta Romanelli (see "Methods" section). This analysis reveals substantial differences in shape between domesticated and wild individuals, and discriminates the specimen R4 from Grotta Paglicci, characterized by reduced crown dimensions, as belonging to a dog while the specimen P6450 from Grotta Romanelli falls in the overlapping area between dogs and wolves (Fig. 4).

**Figure 5:**
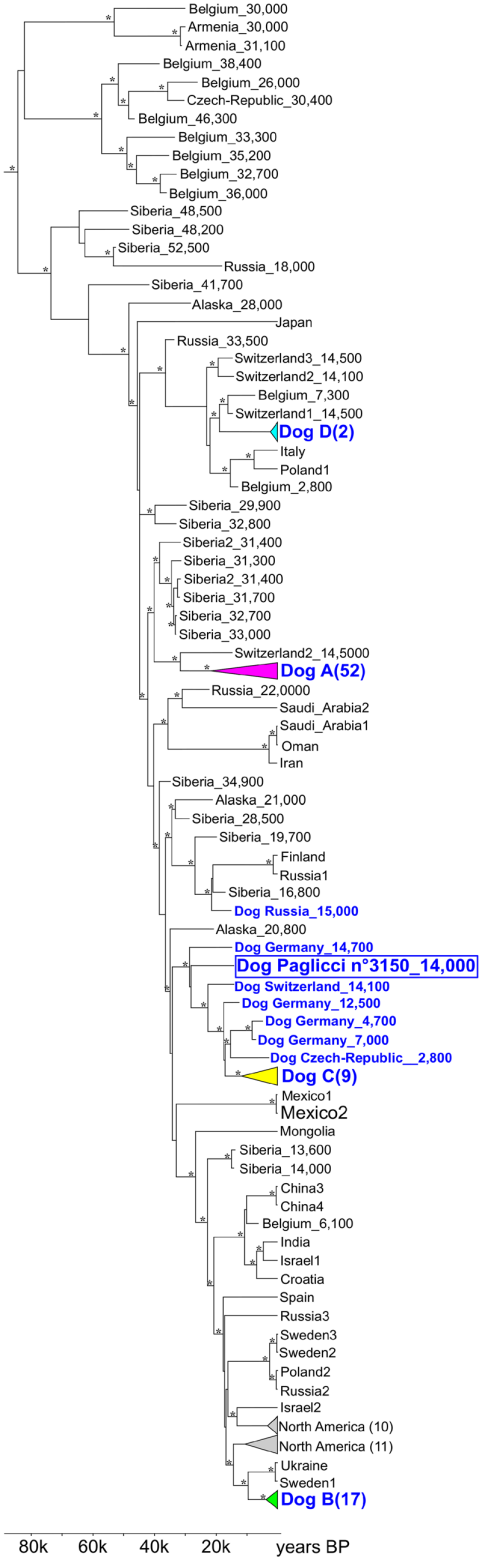
Phylogeny of modern and ancient canids based on mtDNA. The outgroups (three dholes, four coyotes and two Chinese wolf sequences) are not shown. Ancient individuals are labelled with their country of origin and their approximate calibrated ^14^C cal. yr bp age. Ancient dogs are labelled in blue. Monophyletic clusters are collapsed and coloured to highlight the four clades Dog A–D. Number of individuals in each cluster is indicated in brackets. Asterisks highlight nodes with posterior probability > 0.9

We carried out genetic analysis on a small 3rd metatarsal from layer 4c of Grotta Paglicci, sample 3150 (direct ^14^C date: 14,372–13,759 cal. yr bp). A double-stranded library was prepared on the DNA extracted, and target enrichment for the mitochondrial genome was performed, followed by high-throughput sequencing on Illumina platform^50^. The resulting DNA fragments showed typical features of ancient DNA: reduced length with an average of 57 bp and high rates of deamination with 38% of C to T and 48% G to A at the 5′ and 3′ ends of the molecules respectively (Supplementary Table 5 and Supplementary Figure 4). Around 91% of the mitochondrial genome was reconstructed and compared with data from 179 ancient and modern dogs and wolves, 4 coyotes and 3 dholes (see "Methods" section and Supplementary Data S4). A phylogenetic analysis based on a Bayesian approach attributes the specimen from Grotta Paglicci to *Canis lupus* and allows us to exclude its taxonomic attribution to *Cuon alpinus.* The tree (Fig. 5) shows a well-resolved mitochondrial phylogeny with dogs falling within four clades (DOG A–D) as previously described by Thalmann and colleagues^4^. The sample 3150 from Grotta Paglicci is placed in the closest sister group of modern dogs’ clade DOG C. The lineage of the specimen branches off immediately after the dog from Oberkassel (Germany), dated to about 14,800–13,300 cal. yr bp, and before the other ancient dogs from Germany (Karstein dated to 12,500 cal. yr bp, Herxheim dated to 7,000 cal. yr bp and Cherry Tree Cave date to 4,700 cal. yr bp), Switzerland (dated to 14,100 cal. yr bp) and Czech Republic (dated to 2,800 cal. yr bp). The clade that includes the above-mentioned samples is therefore composed exclusively of modern dogs and ancient samples attributed to domestic forms, suggesting that sample 3150 from Grotta Paglicci is a dog. The most recent common ancestor (MRCA) of this clade is estimated to 28,510 cal. yr BP, 95% HPD 25,827–31,314, very close to the MRCA between the lineage of the sample from Grotta Paglicci and the other dogs in clade C (28,048 cal. yr bp, 95% HPD 25,005–30,442).

**Figure 6:**
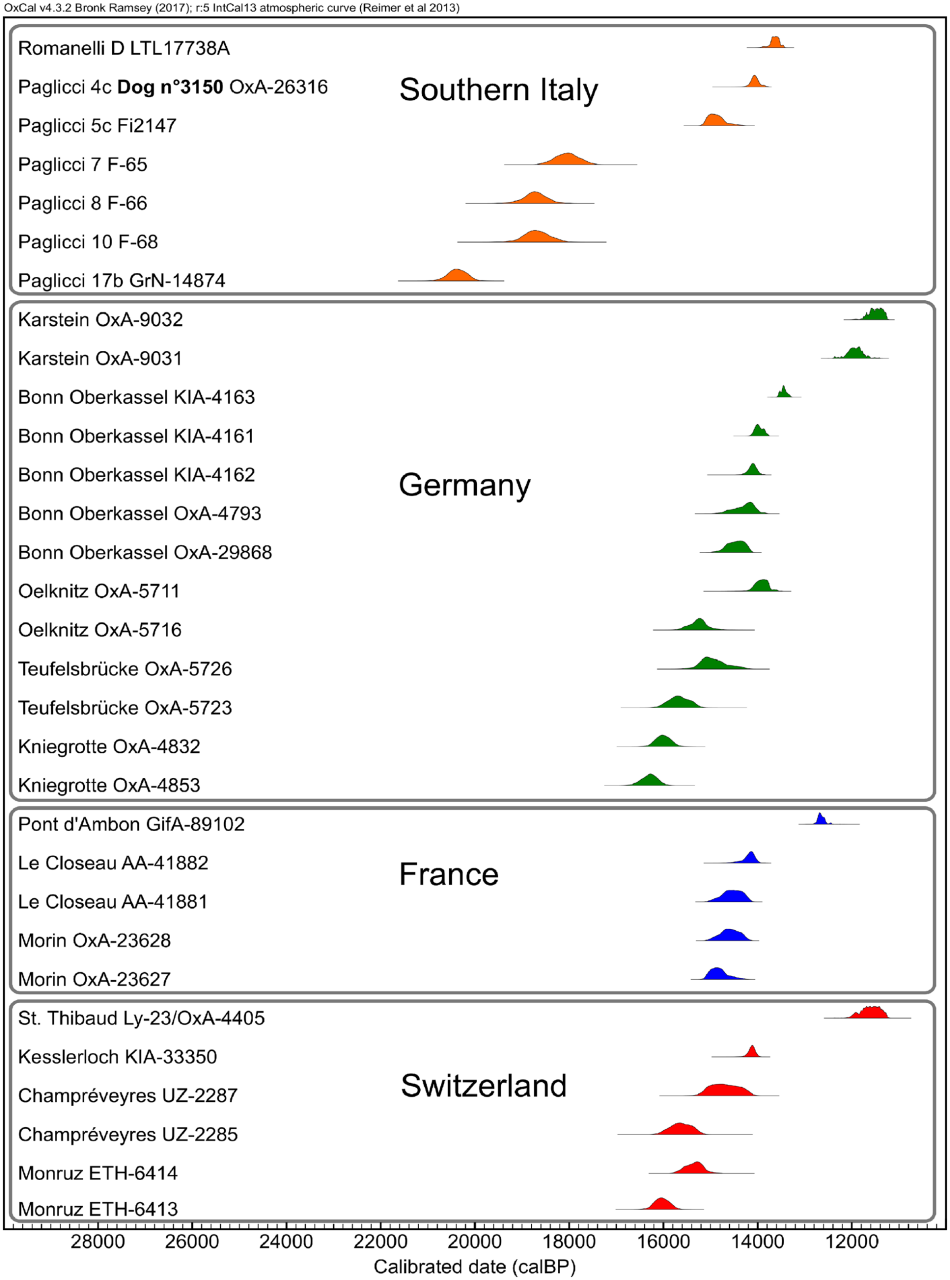
Calibrated ^14^C dates of early European domestic dogs from Apulia compared with other dogs from Europe. Dates of Grotta Paglicci are only related to layers where these remains were found. Layer D of Romanelli is the one where tooth P6450 is from.

Nowadays, very small wolves are observed only in warm and arid contexts^51,52^, which are different to the Last Glacial Maximum and Late Glacial Southern Italy. In addition, the palaeontological record shows that if variations in body size of large canids occurred, populations with significant different body size are mainly stratigraphically or geographically separated^53,54^, whilst the smallest and largest individuals found at Grotta Paglicci are from the same layers (the oldest dated to about 20,000 years ago—GrN-14874; Fig. 6). In addition, as far as we know, the Late Pleistocene bone record from Apulia reveals that OIS 2 small *Canis* individuals are not present in natural accumulations^55–57^, whilst in the cases of Grotta Paglicci and Grotta Romanelli they are associated with human presence. In addition it has to be underlined that this small form shows genetic similarities with dogs at least at 14,000 years ago at Grotta Paglicci, and already acquired dental traits that have to be considered typical for dogs at least at 13,800 years ago at Grotta Romanelli.

Small *Canis* individuals from Grotta Paglicci and Romanelli were about as big as those of the so-called Western European Upper Palaeolithic small dogs group^22^, which differs in size from the (sometimes debated^11^) larger individuals from Eastern Europe and Russia. It is noteworthy that available data highlight similarities between an individual from Paglicci and a German Palaeolithic dog also from a genetic point of view (Fig. 5), possibly suggesting a common origin and a later dispersal across Europe. Our data (body size, genetics and dental internal structure) indicate that dog-like individuals were present in Apulia at least 14,000 years ago and likely as early as 20,000 years ago, as shown by the small dimensions of the phalanx 21865. This suggests that dogs may have represented a common cultural trait among human groups in an historical moment, when a strong cultural diversification occurred, between the Mediterranean world (e.g. the Italian Epigravettian) and the regions north of the Alps (e.g. Magdalenian)^29^.

Our results from archaeological evidence confirm the hypothesis based on genetic models^12^, which constrained the timing of dog domestication to 20,000–40,000 years ago. In addition, the estimated most recent common ancestor between Paglicci and the other dogs of clade C is in agreement with this picture. Some authors consider domestication as related to selection for reduced aggressive behaviour, triggering several physiological and anatomical changes (e.g. size reduction and changes in coat colour, reproductive cycles and hormonal activities)^58,59^; others are more cautious in defining the “domestication syndrome” and consider domestication as a possible result of an adaptation of animal species to a human-modified environment^60^. In this perspective, the presence of *Canis* remains already showing noticeable body changes just after the Last Glacial Maximum can be related to the fact that wolves began to take advantage of a new niche in adverse ecological conditions, becoming human-commensal scavengers. The occupation of this new niche, as well as the subsequent new selective pressure, might have led to a new social ecology^61^ and a new evolutionary response, which triggered domestication. This might have been a key factor in the emergence of a closer relationship between wolves and humans. The earliest small individual (21865) from layer 17 of Grotta Paglicci (Early Epigravettian), appears just after the Last Glacial Maximum, while similar evidence began to appear only some millennia after in central Europe (Germany and Switzerland) and even later in France, eastern Europe, the Middle East and eastern Eurasia. The evidence for “incipient dogs” and “proto-dogs” dated to between about 36,000 and 26,000 years ago, have been criticised^6–11^, and the peculiar cranial morphological features, considered as the proof of domestication, were also found in extinct wolf ecomorphs^62^. Even if some scholars pointed to the possible benefits of cynegetics^23^, behavioural studies on dogs revealed that breeds intensively selected in modern times for carrying out peculiar tasks are more skilled in using human social and communicative behaviour than wolves and “primitive”, less selected breeds^63,64^. It is still controversial whether earliest Palaeolithic dogs were able to interact with humans at a level sufficient to play a key role in subsistence strategies. Anyway, whatever the reason for dogs’ domestication, their presence in Apulia from at least 14,000 years ago and probably as early as 20,000 years ago (Fig. 6) suggests that these animals might have played a critical role in Epigravettian cultures of the region.

## Methods

### Biometry

Measurements of dog and wolf bones from Grotta Paglicci (both), Grotta Romanelli (both), Upper Palaeolithic sites from France (Pont d’Ambon, Montespan, Le Closeau; only dogs)^22^, Grotta delle Ossa (Holocene, Slovenia,only wolves)^65^ and modern wild populations from Portugal^66^ (Supplementary Data S1) were compared with a standard represented by a skeleton of a present adult female wolf individual from Italy stored at the University of Siena (specimen No. 361, shoulder height: 66.7 cm. In order to obtain as much reliable results as possible, we excluded three post-cranial elements from our biometric analysis (R64, from reworked sediments; 7460, not measurable; 17165, possibly shrunk due to combustion). Measurements of skeletal elements are from Von den Driesch^67^ and expressed in mm. The comparative data are detailed in the Supplementary Tables 6, 7, and 8. Data from other extant wild individuals are not used in Fig. 2, because most of studied samples of wolves consist only in skull and mandibles. Out of the six extant wild wolves with available post cranial elements, three are young individuals from Italy (No. 353, 362, and 139), one (No. 138) is another Italian wolf (adult), one is the adult used as the standard and another (LLj) is from Slovenia. We consider that only a few individuals is not enough to represent the variability of a population (neither the Italian, nor the Slovenian one) and we preferred not to add them to Fig. 2. In any case, it does not affect the results and interpretations of this study as fossil wolves that are penecontemporaneous with the fossil dogs studied here are included and are more relevant for comparative purposes.

### Wolf ontogeny

Complete ossification of epiphyses is reached in wolves (and dogs) at about one year of age^46^. To support the idea that wolf long bones showing fused epiphyses belong to individual that already reached at least the minimum adult body size, we analysed body-size data of extant Italian wolves collected on the field by one of the authors (RF). The sample includes 99 individuals from 2 months-old to adult age (Supplementary Data S2). In the Supplementary Figure 5, it is shown that 6 to 10 months-old wolves (thus not mature from a skeletal point of view) already reached a body size comparable with that of older individuals. These results give further support to the hypothesis that small long bones from Grotta Paglicci and Grotta Romanelli belong to adult dogs, rather than to young wolves.

### X-ray microtomography

A total of 61 specimens, including 45 dog and wolf lower first molars, one dog phalanx and 15 first metacarpals (Supplementary Data S3), were analysed by X-ray microtomography (μCT) at the Multidisciplinary Laboratory of the Abdus Salam International Centre for Theoretical Physics (Trieste, Italy), using a system specifically designed for the study of archaeological and paleontological materials^68^. The μCT acquisitions of the specimens were carried out by using a sealed X-ray source (Hamamatsu L8121-03) at variable voltage and current and with a focal spot size of 5 μm (Supplementary Table 9). Sets of 1,440 or 2,400 projections of the samples were recorded over a total scan angle of 360° by a flat panel detector (Hamamatsu C7942SK-25). The resulting μCT slices were reconstructed using the commercial software DigiXCT (DIGISENS) in 32-bit format. Acquisition parameters and the obtained isotropic voxel sizes are reported in the Supplementary Table 9 for all the samples. A semi-automatic threshold-based segmentation was carried out to separate the bone tissue from the interstitial void in post cranial elements, and to separate enamel from dentine^69–71^.

#### First metacarpal

A first metacarpal proximal epiphysis (13247) of a possible dog was identified. Its dimensions are not compatible with those of an adult wolf, but the lack of distal epiphysis does not allow to exclude the presence of a young wolf. A total of 15 wolf and dog first metacarpals were analysed by means of μCT (Supplementary Table 9) to detect the age of the individual 13247 from layer 10D of Grotta Paglicci (Evolved Epigravettian). Comparative sample is composed of three young present-day wild individuals from Italy (353, 362 and 139); three present-day zoo-wolves (52, 180 and 214); three adult present-day wild wolves from Italy and Slovenia (361, 138 and LLj); three wild individuals from Grotta Paglicci (1971, R23 and R24) and two present-day domestic dogs (196 and CLj). After the segmentation, all bones were aligned to their longitudinal axis and the proximal epiphysis was separated from the rest of the bone using a transversal plane tangent to the distal ridge of the articular facet of the second metacarpal (Supplementary Figure 6). The ratio between Bone Volume and Total Volume (BV/TV) was calculated for each epiphysis. Even if the distal epiphysis is already attached (but still not completely fused) with the diaphysis, young not fully developed wolves show a more porous trabecular and cortical bone tissue (i.e. a low BV/TV value) and can be easily separated from the others (Supplementary Figure 6). The specimen 13247 from Paglicci shows a BV/TV compatible only with that of an adult individual of very small size.

#### Burned specimens

In a recent study^45^, some of the authors of the present paper demonstrated that μCT imaging can reveal bone fractures due to shrinkage caused by burning. The analysis of two burned specimens from Grotta Paglicci (21875 and 17165 respectively) showed that a first phalanx from layer 17b (21875) does not show any fracture caused by deformation of bone tissues. The fractures visible in the Supplementary Figure 2 are due to post depositional agents (this specimen was found broken in several fragments during the excavation). The specimen 17165 (a calcined first metacarpal) shows a pattern of fractures within the compact bone tissue clearly compatible with bone deformation (and shrinkage) due to burning. Considering the level of bone shrinkage at high temperatures^72^, its small dimension (distal breadth: 5.2 mm) would not be explained with the reduction of a skeletal element of a wolf-sized individual after burning. Nevertheless, it was excluded from the biometric analysis.

#### Percent of crown dentine in the lower first molar in dogs and wolves

Dentine percent of the lower first molar P6450 from Grotta Romanelli was analysed and compared with that of a sample of 21 dog and 23 wolf specimens (Supplementary Table 4). Among dogs, 18 are present-day individuals whose skeletal remains were collected on the field. The breed is unknown. Three remains, stored at the Natural History Museum of Trieste (NHMT), are archaeological and in particular Holocene individuals coming from the area of Škocjan (Slovenia—old excavations, without clear context). Among wolves, the specimens that are part of the osteological collection of the University of Siena are represented by one present-day zoo-wolf of northern European provenance and by 7 wild individuals coming from central Italy. Other three wild wolves from central Italy are part of the zoological collection of the Fisiocritici Academy of Siena and one is from north-eastern Italy and is part of the zoological collection of the NHMT. Among archaeological wolves, six are Holocene individuals from Grotta delle Ossa (Škocjan, Slovenia, Archaeological collection NHMT)^65^, two are from Grotta Paglicci (one Upper Palaeolithic and one Middle Palaeolithic) and three from Grotta Romanelli (two Upper Palaeolithic and one Middle Palaeolithic). The Middle Palaeolithic specimen from Romanelli comes from the “Terre rosse” level (3596_3, Supplementary Table 4). It was previously considered as belonging to *Canis mosbachensis*, but was recently reassessed to belong to *Canis lupus*^57^. Given that the two Middle Palaeolithic specimens belong to small-bodied wild individuals, these specimens are relevant studied to control the pattern of crown dentine in a small wolves Pleistocene population. Image segmentation of all teeth was carried out using a semi-automatic threshold to separate different dental tissues (dentine and enamel). After adapting our own protocols, developed in virtual dental paleoanthropology^48, 49^, we set a reference tooth cross-section whose orientation was fitted to the cervix. Moving this cross-section across the tooth crown we chose two cross-sections to separate a 3D tooth slice (Supplementary Figure 7). Lower plan (cross-section 1) was set at the bottom of the fossa between the paraconid and the protoconid. The upper plan (cross-section 2) was set at the point where the paraconid and the protoconid separate from each other. We were thus able to carry out a study of the percent of crown dentine in a region corresponding to the middle part of the main cusps. The percent of crown dentine is expressed using the formula: (dentine volume/(dentine volume + enamel volume)) * 100, (Vcdp/Vc (%)). We selected only teeth that do not show wear in this part of teeth. Even if slight, wear is well visible in virtual models as one or more flat facets corresponding to a decrease in enamel thickness (Supplementary Figure 8). Once the obtained results demonstrated that dog lower first molar is characterized by a lower percent of crown dentine (Supplementary Table 4) than in wolves, we applied this methodology to one tooth from Grotta Romanelli (P6450). The tooth is slightly broken at the cervix on the mesial aspect. To correct the missing enamel, we aligned the preserved part of the cervix of the P6450 tooth with those of three other specimens: dog SC1 (archaeological specimen) and two wolves of different size (377 and 6445). In all three cases, when the tooth was oriented and the 3D slice was extracted and analysed, combining the percent of crown dentine with tooth size (breadth) the specimen from Grotta Romanelli falls within dog variability (Fig. 3b).

### The possible presence of ***Cuon alpinus***

The presence in Italy, during the Upper Palaeolithic, of another smaller wild canid, the dhole (*Cuon alpinus*), can be excluded on the basis of palaeontological evidence, since this species disappeared in the Apennine Peninsula during MIS 3^73^. Among the specimens presented in this paper, the distal fragment of a tibia from layer 5a (1632, Final Epigravettian, ca. 15,000 years ago), smaller in size than a homologous fragment of an upper Palaeolithic dog from Pont d’Ambon (France) (specimen 22, table 4 in Pionnier-Capitan et al.^22^) shows a morphology that is not typical of a *Cuon*. In particular, both the prominent edge of malleolus, the rounder and regular margin of the distal articulation in its middle part (anterior view), and a small oblique groove in the lateral half of the distal articular border (anterior view) are well visible and differ from the morphological condition of dholes^22^. In addition, the specimen 3150 from Grotta Paglicci was definitely attributed to a *Canis* by means of palaeogenetics (Fig. 5).

### Lower first molar shape analysis

Employing a geometric morphometric approach, we performed a contour analysis of the outer enamel surface (OES) on a sample of 21 Holocene dogs and 23 Late Pleistocene and Holocene wolves lower first molars (LM1) (Supplementary Table 10). The occlusal plane is defined here as the perpendicular view of a virtual cross-section fitting the cervix. We defined two homologous landmarks in order to constrain the sliding of two curves of 80 and 60 semilandmarks linearly spaced along the outlines (Supplementary Figure 9). Sliding semilandmark method^74^, based on the Procrustes superimposition algorithm, was used for generating shape data^75,76^. We performed generalized Procrustes analyses, principal component analyses (PCA) and between-group principal component analyses (bgPCA) based on the Procrustes shape coordinates^77^. The two canid specimens R4 and P6450 were included a posteriori in the bgPCA. Because one of the teeth (P6450) is slightly broken at the cervix on the mesial aspect, we have made three reconstructions for this specimens, aligning the preserved part of the crown with those of an archaeological dog (SC1), a modern wild wolf (377) and a fossil wolf from Grotta Romanelli (P6445). Then, employing the geometric morphometric approach, we performed the contour analysis. The analyses were performed using the package ade4 v.1.7-6^78^ for R v.3.467. Allometry was tested using multiple regressions^79^ in which the explanatory variable is the centroid size and the dependent variables are the bgPC scores. There is a weak allometric signal along bgPC1 (*p*-value < 0.05; R^2^ = 0.27), and no size-related variation is detected along bgPC2 (*p*-value > 0.05), the differences between specimens in this analysis thus mostly representing shape-variation.

### aDNA analysis

Only the small 3rd metatarsal 3150 from layer 4c of Grotta Paglicci (direct ^14^C date: 14,372–13,759 cal. yr bp) was selected for aDNA analysis. The oldest specimens are too small to extract enough quantity of bone powder without significantly damaging them. DNA analysis was carried out in the Molecular Anthropology Laboratory of the University of Florence, exclusively dedicated to ancient DNA analysis. Blanks as negative controls were used in all of the experimental steps to monitor the absence of contaminants in reagents and environment. To remove potential contamination, the outer layer of the bone was mechanically taken out using a dentist drill with disposable tip. After brushing, sample was irradiated by ultraviolet light for 45 min in a Biolink DNA Crosslinker (Biometra). The DNA was extracted from approximately 50 mg of bone powder following a published silica-based protocol^50,80^ and eluted in 100 µl of TET buffer (10 nM Tris, 1 mM EDTA and 0.05% Tween-20). 20 μl of DNA extract were transformed into genetic library following a double-stranded DNA protocol^81^ using a unique combination of two indexes. Sample and negative controls were checked with Agilent 2100 Bioanalyzer DNA 1000 chip. Libraries were then enriched for mitochondrial DNA following a capture protocol^81,82^ and sequenced on an Illumina MiSeq run for 2 × 75 + 8 + 8 cycles.

#### Bait production

Two overlapping long-range PCR products encompassing the whole mitochondrial canine genome were produced. Primers (Supplementary Table 11) were designed using the Primer3 program (https://frodo.wi.mit.edu/primer3/input.htm). DNA was extracted from the saliva of a special dog of Akita Inu breed and used as template. The PCR purification and subsequent analytical steps to create the baits were carried out following Maricic et al. protocol^50^.

#### Raw reads processing and mapping

The EAGER pipeline^82^ was used for initial sequencing quality control, adapter trimming and paired-end read merging. Only reads with a minimum overlap of 10 bp and with a minimum total length of 30 bp were kept. Filtered reads were mapped to the reference dog mtDNA (U96639)^83^ using BWA-0.7.10^84^ setting recommended parameters for ancient DNA molecules (“-l 1000 -n 0.01 -o 2”)^85^. After mapping, PCR duplicates were removed using SAMtools-1.3.1^86^. Consensus sequence for mtDNA was called using mpileup and vcfutils.pl of the SAMtools package,only the reads with a mapping quality ≥ 30 were used to call confident bases. Finally, we reconstructed the 91.62% of the mitochondrial genome with an average coverage of 2.71 (Supplementary Table 5). Damage patterns were detected using mapDamage2.0^87^: the sample shows a substitution rate at read termini higher than 30%, fully compatible with sample age (Supplementary Table 5 and Supplementary Figure 4). In addition, the low average fragment length (57.62 bp) provides a good indication that the mtDNA obtained is authentic (Supplementary Table 5).

#### Phylogenetic analysis

The assembled mitochondrial genome was used to reconstruct the canine phylogeny together with previously published sequences from 126 modern and 53 ancient dogs and wolves^88^, 4 coyotes and 3 dholes (*Cuon alpinus*) (Supplementary Data S4). Alignment of the mitochondrial genomes was performed by Mega7^89^ with the Muscle algorithm^90^ following criteria proposed in Thalmann et al.^4^. BEAST v2.6.2^91^ was used to determine a phylogenetic tree with Hasegawa-Kishino-Yano and gamma distributed rates (HKY + G) as substitution model, estimated as the best model according to Mega 7^89^. Strict clock model and constant population size were used as priors as suggested in Thalmann et al.^4^ and Skoglund et al.^3^. Tip dates for ancient samples were set according to their radiocarbon calibrated bp ages and used for calibrating and estimating the substitution rate. A MCMC run with 40,000,000 generations, sampling every 2,000 was performed. Effective sampling size (ESS) values and chain convergence were evaluated using Tracer v1.7.1^92^. ESS values were higher than 200 for all the parameters. The first 10% of iterations were discarded as burn-in and TreeAnnotator v2.6.2^91^ was used to produce a Maximum Clade Credibility tree, then visualized by FigTree (https://tree.bio.ed.ac.uk/software/figtree/).

### ^14^C dates

Dates shown in Fig. 6 were calibrated with the software OxCal v.4.3.2^93^ using the IntCal13 curve^94^. Date of Palaeolithic dogs (or relative contexts) from France, Germany and Switzerland are from Street et al.^95^. The radiocarbon date of Grotta Romanelli is from Calcagnile et al.^41^; among dates of Grotta Paglicci, four (GrN-14874, F-65, F-66, F-68) are from Berto et al.^30^ and one (OxA-26316) is a previously unpublished direct date of sample 3150. It was carried out using the dating service of the Oxford Radiocarbon Accelerator Unit. The date was obtained by removing contaminations with a pretreatment^96^. The uncalibrated date in radiocarbon years BP is 12,175 ± 55.

In addition, other new radiocarbon measurements were performed by one of the authors to better contextualize some of the Final Epigravettian layers and to give an age to specimen R4. Analyses were carried out at the Accelerator Mass Spectrometry (AMS) dedicated beam line at LABEC accelerator in Florence (INFN-CHNet, Cultural Heritage Network)^97^. Samples were chemically treated to extract and purify the “good” carbon for the measurement, and finally this carbon was graphitised. Radiocarbon concentration in graphite pellets was obtained by measuring both ^14^C/^12^C and ^13^C/^12^C—to correct for isotopic fractionation—ratios along the beam line; chemistry and accelerator background was evaluated by measuring apparent radiocarbon abundance in blank samples. NIST Oxalic Acid II (SRM 4990C) was used as primary standard, while IAEA C7 was used as secondary standard to check measurement accuracy. Measured radiocarbon ages were calibrated using OxCal software^93^ and IntCal13 calibration curve^94^.

Four samples were selected from Grotta Paglicci to be dated:

- #2269, humerus attributed to a wolf, collected in layer 5C (Final Epigravettian)[98];
- R4, mandible attributed to a canid (see discussion in the text), collected in an Epigravettian reworked layer, whose chronological context was not knowable a priori;
- #2992, radius, attributed to *Cervus elaphus*, collected in layer 4C (Final Epigravettian);
- #5090, second phalanx, attributed to *Equus ferus*, collected in layer 7B (Final Epigravettian).

Bones were treated to extract collagen, the carbonaceous fraction useful for dating. The mineral matrix was completely dissolved by gentle deacidification in HCl solution: a quite mild solution (0.5 M concentration) was employed to reduce the stress for the samples and to maintain the possibility of collagen recovery. The collected organic fraction was cleaned in NaOH solution, to remove possible contamination due to humic substances and it was then converted to gelatin. Unfortunately, the collagen extraction yield was not fully satisfactory. Sample #5090 gave no collagen at all; collagen collected from #2992 was too little to go further with sample preparation procedure. Regarding the sample R4, collagen yield was quite low, below the limit of 1% that is often indicated as a cut-off for good preserved bones, and in fact it gave a poor amount of CO_2_ with respect to the typical samples treated and measured at INFN-LABEC. Considering the possible importance of this sample (no independent indication of the possible date of this sample was present), the collected CO_2_ was anyway converted to graphite, adding a known amount of carbon dioxide produced by the combustion of a blank sample, in order to have a total mass of graphite as much uniform as possible. Sample #2269 gave a satisfied collagen recovery yield, so that two pellet samples were prepared. This gave us the possibility to check for the presence of possible contaminations. The Supplementary Table 2 summarizes the results of AMS measurements. The two graphite samples prepared from #2269 gave results that were consistent within the experimental error, thus the best estimation of its radiocarbon concentration was obtained as the weighted average of the two measured concentration. The conventional radiocarbon age and thus the calibrated age was derived accordingly (Supplementary Table 2).

The relatively high experimental uncertainty on the measured radiocarbon concentration of R4 is basically given by the experimental error on the CO_2_ pressure measurement in the graphitization reactor.

### Provenance of archaeological remains studied in this paper

*Canis* remains from Grotta Paglicci were excavated by the University of Siena (Dipartimento di Scienze Fisiche, della Terra e dell’Ambiente) with permission of the local Heritage Office (Soprintendenza Archeologia, Belle Arti e Paesaggio per le Province di Barletta – Andria – Trani e Foggia). Remains from Grotta Romanelli are stored in the Bioarchaeology Section of Museo delle Civiltà, Museo Nazionale Preistorico Etnografico “Luigi Pigorini” and were studied with permission from the same Institution. Remains from Škocjan (Slovenia) are part of the collections of the Natural History Museum of Trieste and were studied with permission from the same Institution.

## Supplementary information

Supplementary Information 1.

Supplementary Information 2.

Supplementary Information 3.

Supplementary Information 4.

Supplementary Information 5.

## Data Availability

In regards of genetic data, the consensus sequence is available at the National Center for Biotechnology [GeneBank Accession Number: MH376892]. The other datasets generated during and/or analysed during the current study are available from the corresponding author on reasonable request.
