# Supplementary material for: The first evidence for Late Pleistocene dogs in Italy
Source: Sci Rep. 2020 Aug 7;10:13313. doi: 10.1038/s41598-020-69940-w (PMC7414845; doi:10.1038/s41598-020-69940-w)
Supplement: Supplementary file 1 — Supplementary Information 1. [file 41598_2020_69940_MOESM1_ESM.pdf]

The first evidence for Late Pleistocene dogs in Italy

Francesco Boschin[1*], Federico Bernardini[2,3†], Elena Pilli[4,†], Stefania Vai[4†], Clément Zanolli[5†], Antonio Tagliacozzo[6], Rosario Fico[7], Mariaelena Fedi[8], Julien Corny[9], Diego Dreossi[10], Martina Lari[4], Alessandra Modi[4], Chiara Vergata[4], Claudio Tuniz[3,2,11], Adriana Moroni[1,12,13], Paolo Boscato[1], David Caramelli[4], Annamaria Ronchitelli[1].

[1]Università degli Studi di Siena, Dipartimento di Scienze Fisiche, della Terra e dell'Ambiente, U.R. Preistoria e Antropologia, Via Laterina 8, 53100 Siena, Italia
[2]Centro Fermi-Museo Storico della Fisica e Centro di Studi e Ricerche Enrico Fermi, Piazza del Viminale 1, 00184 Roma, Italia
[3]Multidisciplinary Laboratory, The Abdus Salam International Centre for Theoretical Physics, Via Beirut 31, 34151, Trieste, Italia
[4]Department of Biology, University of Florence, Laboratory of Anthropology -Molecular Anthropology and Forensic Unit, Firenze, Italia
[5]Laboratoire PACEA, UMR 5199 CNRS, Université de Bordeaux, Bâtiment B8, allée Geoffroy Saint Hilaire, 33615 Pessac Cedex, France
[6]Bioarchaeology Section of Museo delle Civiltà, Museo Nazionale Preistorico Etnografico "Luigi Pigorini", Piazza G. Marconi 14, 00144 Roma, Italia
[7]Centro di Referenza Nazionale per la Medicina Forense Veterinaria, Istituto Zooprofilattico Sperimentale delle Regioni Lazio e Toscana "M. Aleandri" Viale Europa, 30 58100 Grosseto, Italia
[8]INFN (Istituto Nazionale di Fisica Nucleare) Sezione di Firenze, via Sansone 1, 50019 Sesto Fiorentino (Fi), Italia
[9]Département Homme & Environnement, Muséum National d'Histoire Naturelle, UMR 7194, CNRS, Musée de l'Homme, Paris, France
[10]Sincrotrone Trieste S.C.p.A., AREA Science Park, Basovizza (Trieste), Italia
[11]Centre for Archaeological Science, University of Wollongong, Northfields Avenue, Wollongong, New South Wales, 2522, Australia
[12]Centro Studi sul Quaternario Onlus, Sansepolcro, Arezzo, Italy
[13]Istituto Italiano di Paleontologia Umana, Roma, Italy

*Corresponding author: Francesco Boschin, fboschin@hotmail.com

[†]these authors contributed equally to the manuscript

Supplementary Information

This PDF file includes:

Supplementary text

Figs. S1 to S9

Tables S1 to S11

Other supplementary materials for this manuscript include the following files:

Datasets S1 to S4

Supplementary Material

Specimens from Grotta Paglicci and Grotta Romanelli whose attribution to domesticated individuals is discussed in the paper are described below and listed in the Supplementary Table 1. Specimens from Paglicci were not discussed in the last zooarchaeological papers on the site[31,98] and are presented here for the first time.

Specimens from Grotta Paglicci:

3150, layer 4c – Third metatarsal, right side. The proximal end is lacking, the distal epiphysis is fused.

3151, layer 4c – Third metatarsal, left side. The distal epiphysis is fused.

1632, layer 5a – Tibia, left side. Only the fused distal epiphysis is preserved.

2053, layer 5b – First phalanx, digit II or V. The proximal epiphysis is fused.

1566, layer 5b – Second phalanx, digit III or IV. The proximal epiphysis is fused.

5110, layer 7b – Second phalanx, digit II or V. The proximal epiphysis is fused.

7460, layer 8b, near the cave's wall – First phalanx, only the distal end is preserved.

13427, layer 10d. First metacarpal, left side. Only the proximal end is preserved.

17165, layer 11a. First metacarpal, right side. Only the distal epiphysis (fused) is preserved and the bone is calcined.

21865, layer 17b. First phalanx, digit II or V. Only the fused proximal epiphysis is preserved. The bone is carbonized.

R64, reworked area. Atlas.

R4, reworked area, mandible, left side. Only a part of the ramus is preserved. Even if the size of the molar row fits the variability of wolves (length of molar row: 43.7 mm), the lower first molar is of reduced size (GL: 25.6 mm).

Specimens from Grotta Romanelli:

P6450, Terre Brune – Lower first molar, left side. The main cusps are slight abraded; the anterior part of the tooth's crown and a part of the anterior root are missing.

6453, Terre Brune – Fourth metacarpal, left side. The distal epiphysis is fused.

5788, Terre Brune – First phalanx, III or IV digit. The proximal epiphysis is fused.

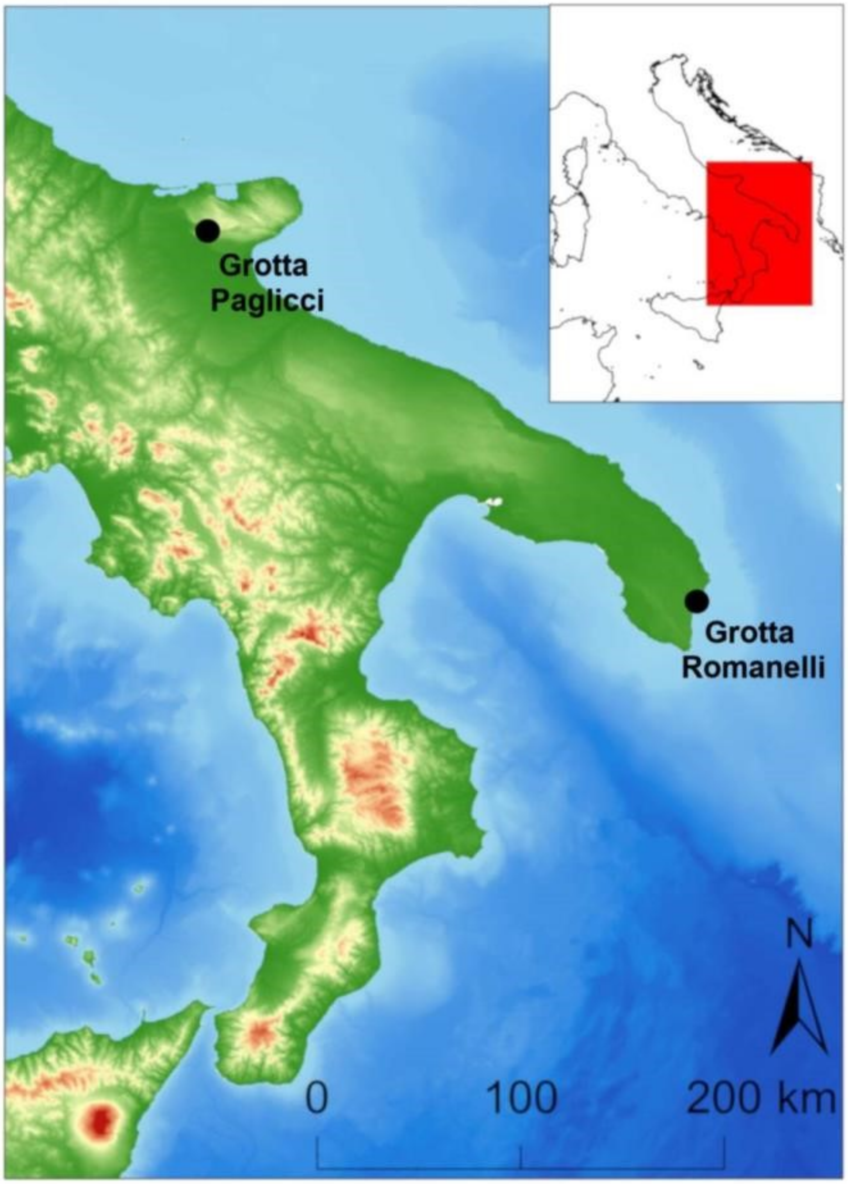
Grotta
Paglicci
Grotta
Romanelli
N
0
100
200 km

Supplementary Figure 1 | Location of Grotta Paglicci and Grotta Romanelli in Italy. Map created with ArcGIS® 10.7.1. Source of Digital Elevation Model: Copernicus Land Monitoring Service – EU-DEM (https://www.eea.europa.eu/data-and-maps/data/copernicus-land-monitoring-service-eu-dem). Source of Bathymetric Model: European Marine Observation and Data Network – EMODnet (https://www.emodnet-bathymetry.eu/data-products). Source of Europe coastline: European Environment Agency – EEA (https://www.eea.europa.eu/data-and-maps/data/eea-coastline-for-analysis-2).

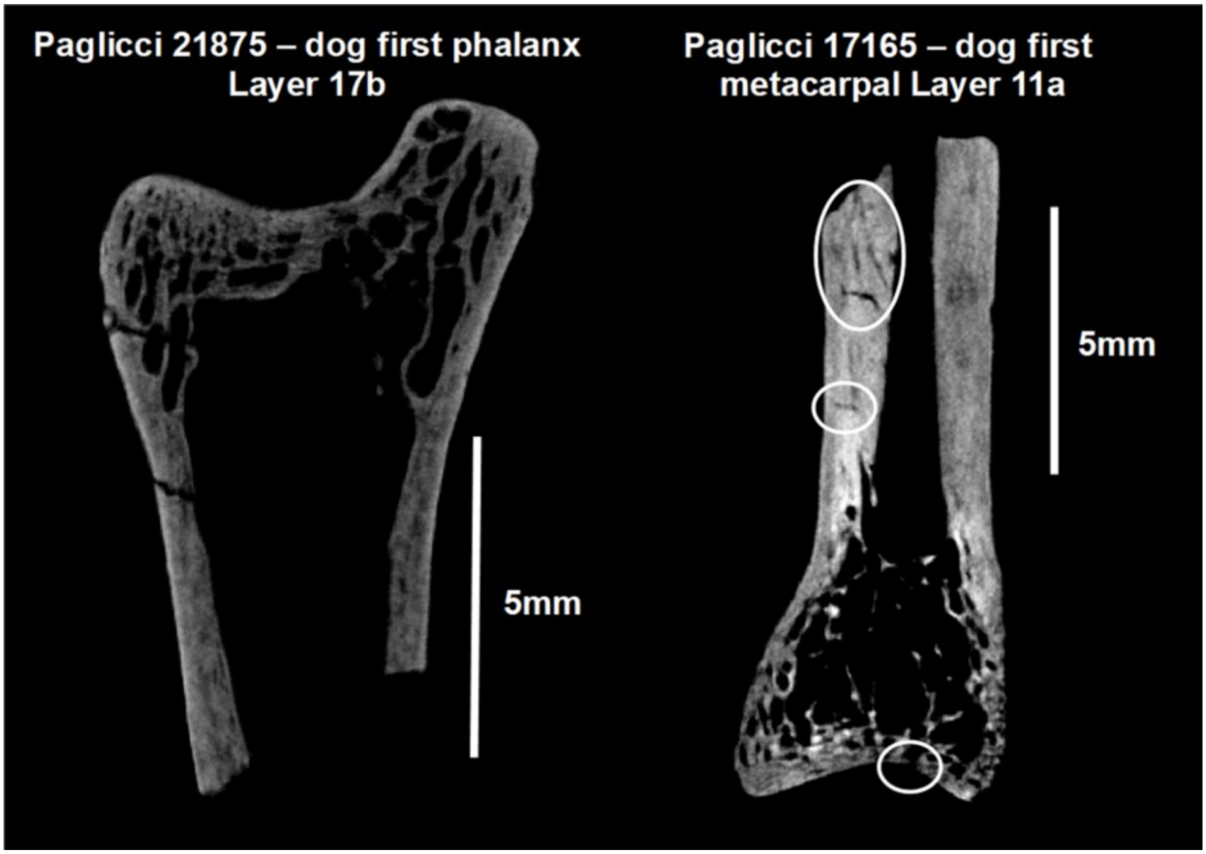

Supplementary Figure 2. | Longitudinal cross sections of the two dog burned specimens from Grotta Paglicci. White circles indicate cracks caused by bone deformation during burning.

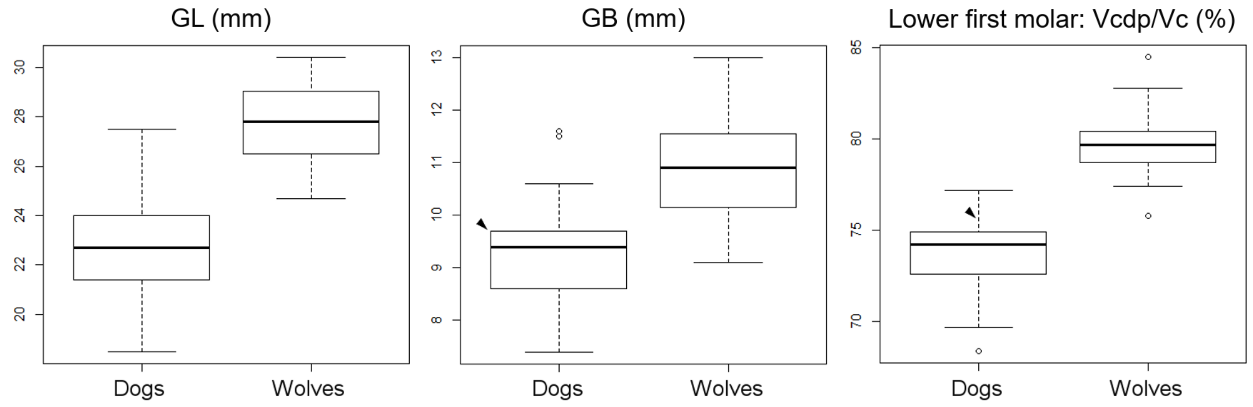

Supplementary Figure 3 | Lower first molar dimensions and tissue proportions. Distribution of values of length (GL; mm), breadth (GB; mm) and crown dentine proportion (Vcdp/Vc; %) in dogs and wolves (data in Table S7). The black arrows indicate the position of specimen P6450 from Grotta Romanelli.

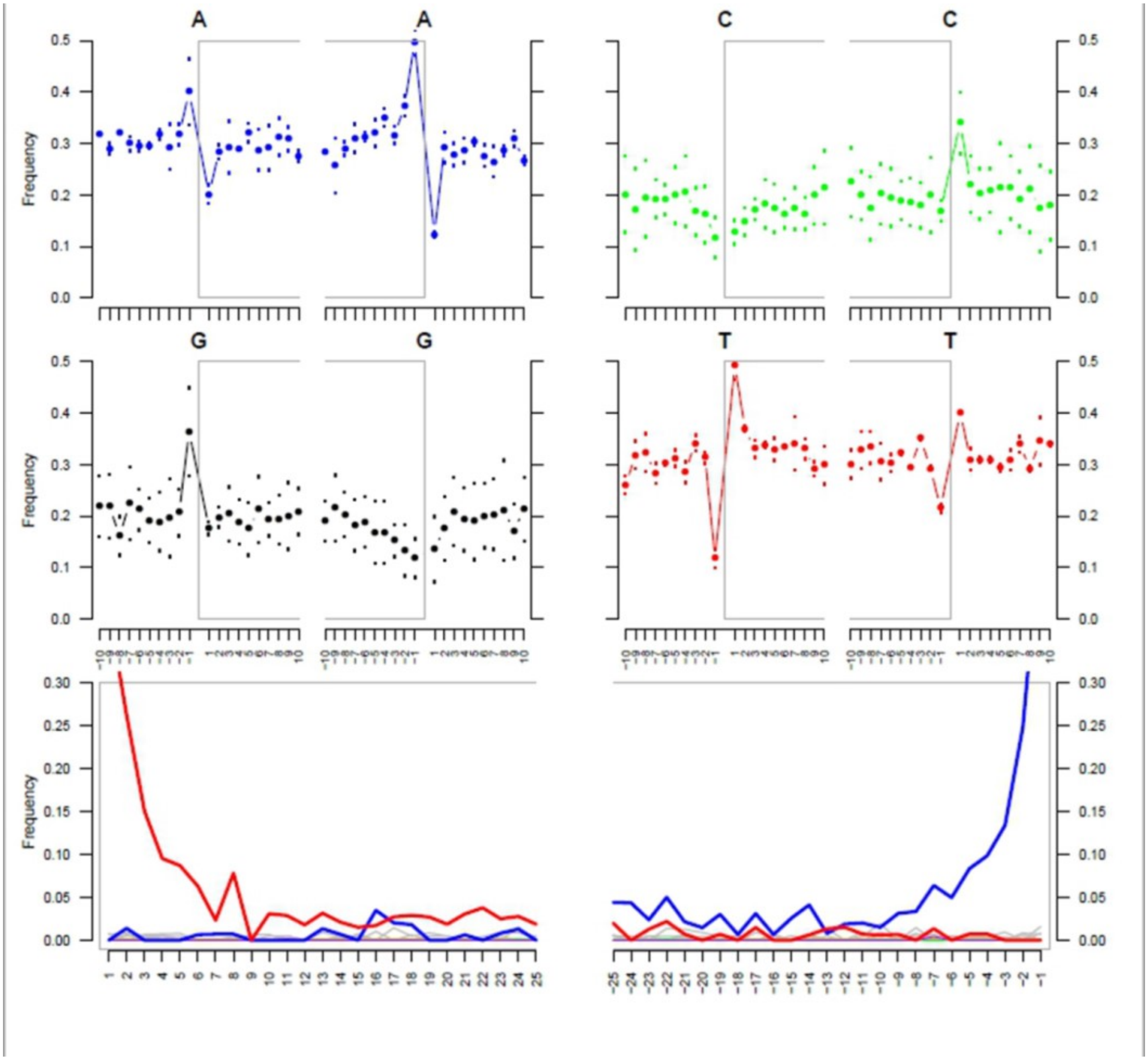

Supplementary Figure 4 | Misincorporation pattern for the sequences mapping on the reference DNA. The CtoT deamination rate at the 5' end is shown with a red curve on the left, the GtoA deamination rate at 3' end is shown with a blue curve on the right.

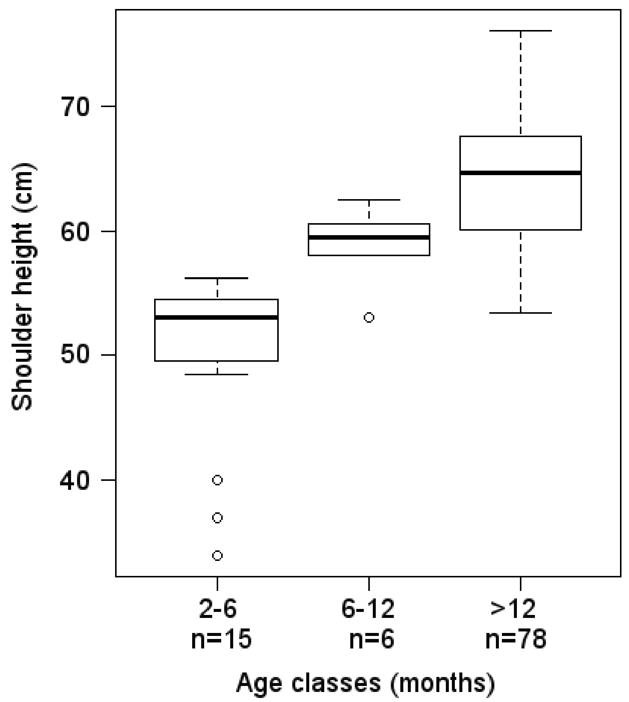

Supplementary Figure 5 | Shoulder height of present-day Italian wolves according to age classes.

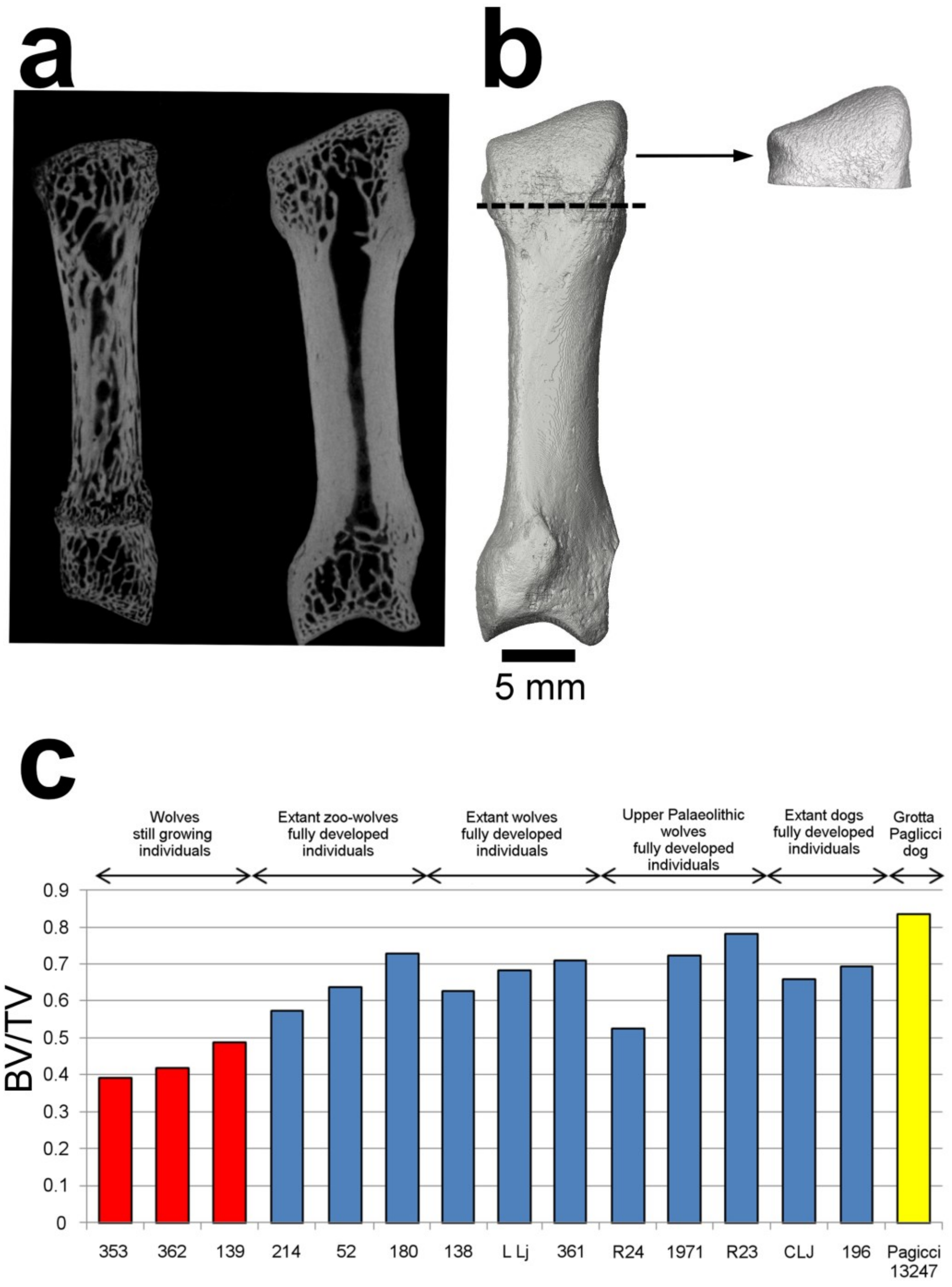

Supplementary Figure 6 | μCT analysis of first metacarpals. a, longitudinal cross-sections of specimens 362 (on the left, juvenile) and 361 (on the right, adult). **b,** virtual extraction of proximal epiphysis from a first metacarpal (n° 361). **c,** BV/TV ratios of analysed first metacarpal proximal epiphyses.

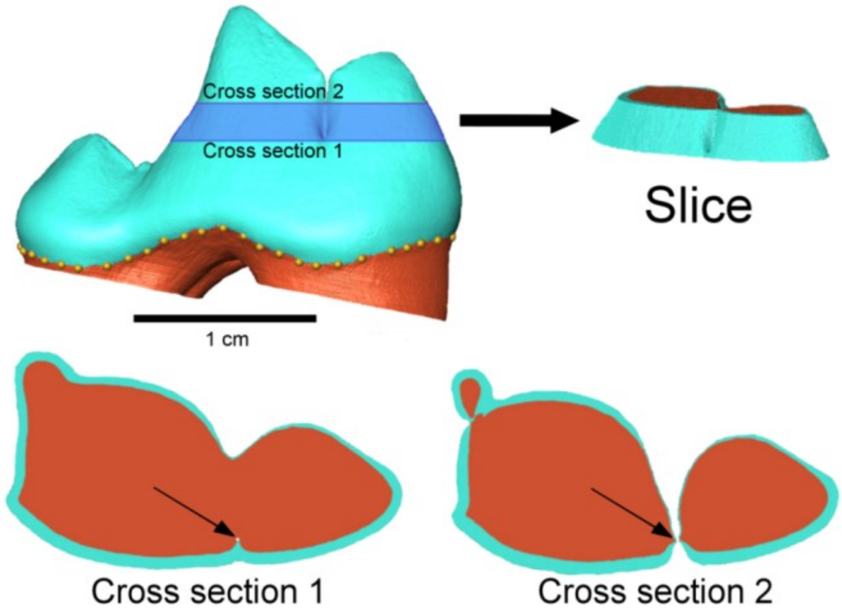

Supplementary Figure 7 | 3D tissue proportions in the lower first molar. In order to avoid the influence of occlusal wear, only teeth showing minimal wear were selected and the volume of enamel and dentine comprised between the cross-sections 1 and 2 (bottom row) was extracted to measure tissue proportions. The Black arrow in cross section 1 indicates the bottom of the fossa between the paraconid and the protoconid. Black arrow in cross section 2 indicates the point where the paraconid and the protoconid separate from each other (see Methods).

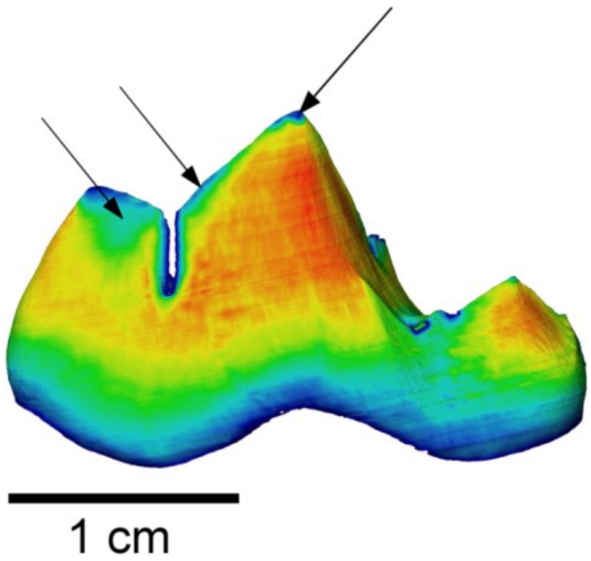

Supplementary Figure 8 | Enamel thickness distribution on a wolf lower first molar (specimen Vpa6312). Enamel thickness increases from thin (blue) to thick (red). Black arrows indicates the presence of wear facets.

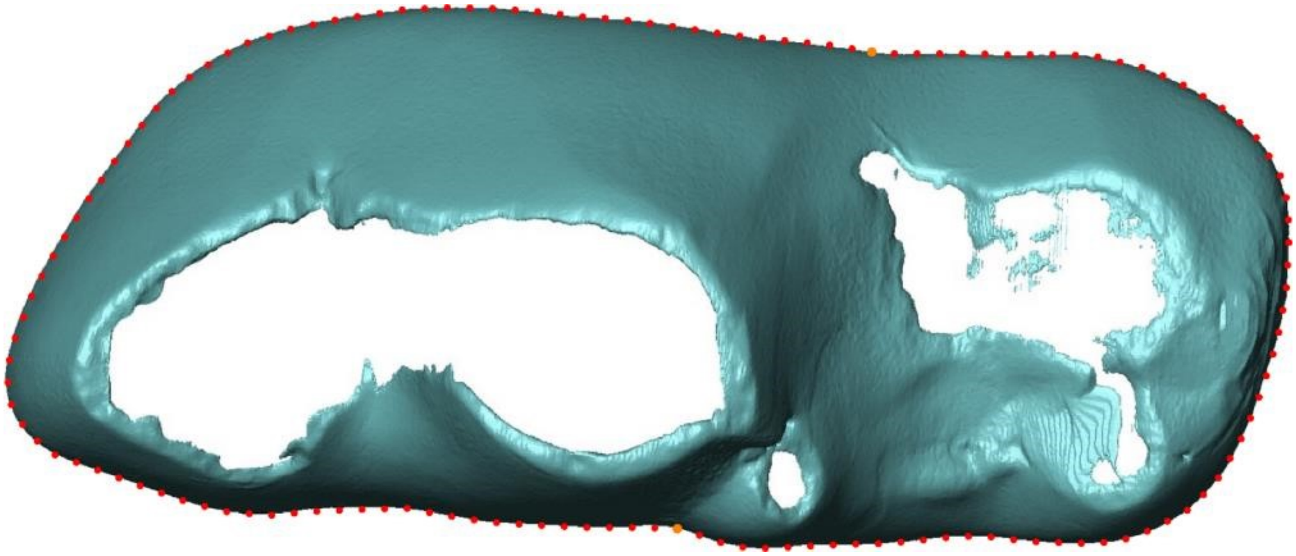

Supplementary Figure 9 | Position of the landmarks (orange points) and semilandmarks. Two semilandmark curves are placed on the crown outline of the lower first molar of the specimen R4 from Grotta Paglicci.

Supplementary Table 1 | List of the Upper Palaeolithic canid specimens from Grotta Paglicci and Grotta Romanelli discussed for their taxonomic attribution.

Grotta Paglicci			Measurements										Taxonomy
N°	Layer	Element	GL	Bp	Dp	SD	Bd	Bfd	Dd	Bfcd	Lad	H	
3150	4c	Metatarsal III				6.8	9.9						Dog
3151	4c	Metatarsal III	70.5		14	6.9	9.5	8.4	9.4				Dog
1632	5a	Tibia					22.1		18.2				Dog
1566	5b	Phalanx 2(III-IV)	19.7	7.5		5	7.5						Dog
2053	5b	Phalanx 1(II-V)	22.8	8.5		4.6	6.9						Dog
5110	7b	Phalanx 2(II-V)	15.5	8.6		6.5	8.8						Dog
7460	8b/wall	Phalanx 1					7.1						Dog
13247	10d	Metacarpal I		6									Dog
17165	11a	Metacarpal I					5.2						Dog
21865	17b	Phalanx 1 (II-V)		7.6									Dog
R64	Reworked	Atlas	31.1							25.4	11.6	21.7	Dog
Grotta Paglicci			Measurements										Taxonomy
N°	Layer	Element	10	13	13a	14	15	16					
R4	Reworked	Mandible	43.7	25.6	10.8	24.7	12.2	6.3					dog
Grotta Romanelli			Measurements										Taxonomy
N°	Layer	Element	GL	Bp	SD	Bd	SDO	DPA	LAR	GL	GB		
P6453	Terre	Metacarpal	63.1			8.5							Dog

	brune	IV											
P5788	Terre brune	Phalanx I (III-IV)	25	8.9	5.9	7.7							Dog
P6450	Terre brune	M/1								24.4	9.7		Dog

Supplementary Table 2 | New radiocarbon AMS dates. Calibration was obtained through OxCal software, considering the IntCal13 calibration curve.

Sample	Layer	Lab code	^{14}C concentration (pMC)	Radiocarbon age (BP)	Calibrated age (cal. yr BP) – 68% prob.	Calibrated age (cal. yr BP) – 95% prob.
R4	Reworked	Fi2146	24.6 ± 3.4	11300 ± 800	14400 – 12100	15800 – 11200
#2269	5c	Fi2147	21.09 ± 0.22	12570 ± 60	15100 – 14750	15200 – 14450
		Fi2148	20.72 ± 0.22			

Supplementary Table 3 | Standard measurements for wolf skeletal elements.

Element	GL	GB	Bp	SD	Bd	SLC	DPA	SDO	DC
**Scapula**						30.1			
**Humerus**					41				36.4
**Radius**			22.5						
**Ulna**							30.7	26.4	
**Metacarpal I**	26.1		7.8		7				
**Metacarpal II**	69.6			8.3	11.2				
**Metacarpal III**	80.4			7.2	11.3				
**Metacarpal IV**	78.9			7.8	11				
**Metacarpal V**	67			8.5	11.5				
**Femur**			46	15.2					22.6
**Tibia**					26.8				
**Talus**	31.9								
**Calcaneum**	54.1	22.8							
**Metatarsal II**	76.2			7.4	10.7				
**Metatarsal III**	85.4			8.2	11				
**Metatarsal IV**	87.7			6.5	10.6				
**Phalanx 1 (II-V)**	27.8		11	6.8	9				
**Phalanx 1 (II-V)**	28.5		10.5	7.1	9				
**Phalanx 1 (II-V)**	28		10	6.5	8.5				
**Phalanx 1 (II-V)**	28.5		9.8	5.8	8.3				
**mean**	28.2		10.3	6.5	8.7				
**Phalanx 1 (III-IV)**	31.7		10.6	6.8	9.1				
**Phalanx 1 (III-IV)**	31.9		10.4	6.8	9.2				
**Phalanx 1 (III-IV)**	32.5		10.3	6.5	8.8				
**Phalanx 1 (III-IV)**	33.1		10.3	6.5	8.6				

**mean**	32.3		10.4	6.65	8.9				
**Phalanx 2 (II-V)**	17.3		9.2	6.5	8.6				
**Phalanx 2 (II-V)**	17.4		8.8	6.2	8.1				
**Phalanx 2 (II-V)**	17.1		9.5	6.9	9.1				
**Phalanx 2 (II-V)**	18		9.5	7.2	9.2				
**mean**	17.4		9.2	6.7	8.7				
**Phalanx 2 (III-IV)**	22.6		9	6	8.5				
**Phalanx 2 (III-IV)**	22.6		9.2	6	9				
**Phalanx 2 (III-IV)**	21.5		9.7	6.7	9.4				
**Phalanx 2 (III-IV)**	22.1		9.7	6.6	9.6				
**mean**	22.2		9.4	6.3	9.1				

Supplementary Table 4 | Measurements of the lower first molars P6450 compared with Holocene dogs and Holocene and Pleistocene wolves. Measurements of great length (GL) and great breath (GB) are from von den Driesch[65], whereas the lateral percent of crown dentine (Vcdp/Vc) was assessed in this study (see Methods).

**Taxon**	**Specimen**	**Geographic context**	**GL**	**GB**	**Vcdp/Vc (%)**
Upper Palaeolithic *Canis*	P6450	Grotta Romanelli (oriented with 377)		9.7	76.52
Upper Palaeolithic *Canis*	P6450	Grotta Romanelli (oriented with P6445)		9.7	76.8
Upper Palaeolithic *Canis*	P6450	Grotta Romanelli (oriented with SC1)		9.7	77,01
dog – present-day	1	Italy	22.4	8.8	68.4
dog – present-day	2	Italy	22.2	8.6	69.7
dog – present-day	70	Italy	21.3	9.1	74.6
dog – present-day	196	Italy	25.3	11.6	73.0
dog – present-day	757	Italy	27.5	11.5	75.5
dog – present-day	1359	Italy	21.4	9	72.6
dog – present-day	95F	Italy	24	9.6	77.2
dog – present-day	Chiostraccio	Italy	20.5	7.9	74.5
dog – present-day	M766	Italy	25.2	10.3	72.9
dog – present-day	TS nonum	Italy	19.9	8.1	71.3
dog – present-day	TS10	Italy	23	9.3	76.3
dog – present-day	TS11	Italy	25	10	75.7
dog – present-day	TS13	Italy	18.5	7.4	74.2
dog – present-day	TS3	Italy	22	9.7	74.9
dog – present-day	TS6	Italy	22.7	9.7	71.9
dog – present-day	TS7	Italy	23.5	9.7	73.9
dog – present-day	TS8	Italy	25.6	10.6	72.9
dog – present-day	TS9	Italy	23.8	9.4	74.4
dog - archaeological – Holocene	SC1	Škocjan - Slovenia	19.8	8	71.8
dog - archaeological – Holocene	SC3	Škocjan - Slovenia	21.4	8.6	74.9
dog - archaeological – Holocene	Vpa6831	Škocjan - Slovenia	23.2	9.5	74.4

Wolf - present-day (zoo)	52	Northern Europe	30	12.1	77.8
Wolf - present-day (wild)	353	Central Italy – University of Siena	27.3	10.1	79.2
Wolf - present-day (wild)	358	Central Italy – University of Siena	28	10.8	79.1
Wolf - present-day (wild)	359	Central Italy – University of Siena	28.6	10.8	84.5
Wolf - present-day (wild)	375	Central Italy – University of Siena	26.4	10	78.5
Wolf - present-day (wild)	376	Central Italy – University of Siena	29.5	11.2	79.7
Wolf - present-day (wild)	377	Central Italy – University of Siena	27	10.2	81.1
Wolf - present-day (wild)	378	Central Italy – University of Siena	27.5	10.2	78.9
Wolf - present-day (wild)	551	North eastern Italy (NHMT)	25	11.1	79.7
Wolf - present-day (wild)	fis_135	Central Italy (Fisiocritici Academy)	26.5	10	80.2
Wolf - present-day (wild)	fis_136	Central Italy (Fisiocritici Academy)	28.3	11.5	80.9
Wolf - present-day (wild)	fis_139	Central Italy (Fisiocritici Academy)	26.5	10.51	78.9
Wolf - archaeological - Holocene	Vpa6312	Škocjan - Slovenia	28.4	10	78.4
Wolf - archaeological - Holocene	Vpa6296	Škocjan - Slovenia	28	11.6	75.8
Wolf - archaeological - Holocene	Vpa6302	Škocjan - Slovenia	30.3	12.3	79.7
Wolf - archaeological - Holocene	Vpa6303	Škocjan - Slovenia	27.7	11	79.8
Wolf - archaeological - Holocene	Vpa6304	Škocjan - Slovenia	27.8	11	80.1
Wolf - archaeological - Holocene	Vpa6309	Škocjan - Slovenia	29.8	12.1	82.8
Wolf - archaeological - Middle Palaeolithic	877	Grotta Paglicci	26.4	10.9	80.7
Wolf - archaeological - Middle Palaeolithic	3596_3	Grotta Romanelli	24.7	9.1	82.1
Wolf - archaeological - Upper Palaeolithic	P6265	Grotta Romanelli	30.4	13	77.6
Wolf - archaeological - Upper Palaeolithic	P6445	Grotta Romanelli	25.7	10	79.5
Wolf - archaeological - Upper Palaeolithic	R38	Grotta Paglicci	29.8	11.7	77.4

Supplementary Table 5 | Sequencing and bioinformatic analyses of Paglicci 14000. Summary including the number of raw reads, number of merged reads, number of mapped reads before and after removing PCR duplicates, depth of coverage, percentage of deamination at the end of the molecules and average fragment length.

Sample ID	Raw reads (R1 and R2)	Merged reads (% of merged)	Mapped reads prior RMDup	Mapped reads after RMDup	Mean coverage	5' Deamination (%)	3' Deamination (%)	Average fragment length (bp)
Paglicci_14000	1,821,888	947,682 (88.18)	2,643	707	2.71	37.86	47.71	57.62

Supplementary Table 6 | Upper Palaeolithic wolf measurements from Grotta Paglicci (known stratigraphic context).

Grotta Paglicci			Measurements										
**N°**	**Layer**	**Element**	**GL**	**GB**	**Bp**	**SD**	**Bd**	**DC**	**Bfcr**	**SBV**	**LCDe**	**10**	**14**
**3470**	4 B	M/2	12	8.6									
**16515**	10 C	M/2	11	8.3									
**19337**	12 F	M/3	6.3	6									
**14706**	10 E3	M\1	15.9	19.4									
**17775**	12 D	M/1	31.3	12.7									
**12598**	9 B1	M/1	30.1	12.8									
**12598**	9 B1	M/2	12.2	7.7									
**12598**	9 B1	M/3	5.8	5.4									
**12598**	9 B1	Mandible										45.3	29.7
**14486**	10 E3	Axis							40.5	28.4	55		
**2269**	5 C	Humerus					47.7						
**17472**	12 A2	Metacarpal II-V					11.3						
**2531**	5 C	Femur			44.8			22					
**10518**	16A1	Metacarpal IV			10.6								
**1632**	5 A	Tibia			22.4								
**604**	17 H	Calcaneum	54.8	22									
**3150**	4 C	Metatarsal III					9.9						
**3151**	4 C	Metatarsal III	70.5				9.5						
**12687**	9 B1	Metatarsal	83.6			7	10.6						
**20177**	12 G	Phalanx 1 (III-IV)			11.7	7.7							
**14670**	10 E3	Phalanx 1 (III-IV)					9.4						
**12641**	9 B1	Phalanx 1 (III-IV)			11.3	6.4							
**12009**	10 E1	Phalanx 1 (III-IV)	34.3		10.9	7.2	9.6						
**14229**	10 E	Phalanx 1 (II-V)	31.1		10.9	6.8	9.5						
**15067**	10 E4	Phalanx 1 (II-V)	28.2		11.2	7.7	9.2						
**16422**	10 C	Phalanx 1 (II-V)	30.4			7.4	9.5						
**16888**	11 B	Phalanx 1 (II-V)	27.4		11.3	7.4	9.7						
**11755**	16 C1	Phalanx 1 (III-IV)	35.6		11.4	7.2	9.3						
**21930**	17 F	Phalanx 1 (III-IV)	39		12.2	8.1	10.6						

**2895**	4 A	Phalanx 2 (II-V)	18.4		10.1	6.5	8.8						
**2515**	5 C	Phalanx 2 (II-V)	17.5		10	7.9	10.1						
**16217**	10 B	Phalanx 2 (II-V)	18.1		10	7.3	9.4						
**17001**	11 B	Phalanx 2 (II-V)	17.4		10	7.4	9.8						
**1692**	23 A	Mc II	75.9				12.9						
**1689**	23 A	Mc III	86.5				10.7						
**1688**	23 A	MC V	73.2				13						
**1690**	23 A	Mt III	94.8				10.6						
**825**	23 A	Mt V	85.4				11.2						
**37**	23 A	Tibia	232				27.9						

Supplementary Table 7 | Upper Palaeolithic wolf measurements from Grotta Paglicci (Epigravettian reworked stratigraphic context).

Grotta Paglicci			Measurements													
N°	Layer	Element	GL	GB	Bp	SD	Bd	DC	SLC	DPA	SDO	Bfcr	Bfcd	GLF	H	10
**R11**	Layers 17-20	Mandible														42.9
**R11**	Layers 17-20	M/1	27.3	11.2												
**R38**	Reworked - Epigravettian	M/1	29.8	11.7												
**R37**	Reworked - Epigravettian	Atlas													27.7	
**R40**	Reworked - Epigravettian	Atlas										45.4			34	
**R12**	Reworked - Epigravettian	Scapula							33.6							
**R34**	Reworked - Epigravettian	Humerus					39									
**R17**	Reworked - Epigravettian	Radius			23											
**R58**	Reworked - Epigravettian	Ulna								36.6	30					
**R23**	Reworked - Epigravettian	Metacarpal I	30		7.7											
**R24**	Reworked - Epigravettian	Metacarpal I	29.4		8.7											
**R62**	Reworked - Epigravettian	Metacarpal IV					13.1									
**R42**	Reworked - Epigravettian	Metcarpal V					12.8									
**R61**	Reworked - Epigravettian	Metacarpal V					12.5									
**R63**	Reworked - Epigravettian	Femur			51.2	16.8		24								
**R2**	Reworked -	Talus	30.7													

	Epigravettian															
**R3**	Reworked - Epigravettian	Calcaneum	55.8	22.3												
**R55**	Reworked - Epigravettian	Calcaneum	59.4	24.3												
**R56**	Reworked - Epigravettian	Calcaneum	60.5	24.2												
**R60**	Reworked - Epigravettian	Metatarsal III	92.7			8.2	11.1									
**R59**	Reworked - Epigravettian	Metatarsal IV				7.5	10.2									
**R22**	Reworked - Epigravettian	Metapodial III-IV	88.6				12.7									
**R8**	Layers 6-10?	Phalanx 1 (II-V)	31.2		12.6	7.8	10.4									
**R9**	Reworked - Epigravettian	Phalanx 1 (III-IV)	34.8		11.1	7.9	9.7									
**R10**	Reworked - Epigravettian	Phalanx 1 1 (III-IV)	31.1		10.4	6.6	9.2									
**R16**	Reworked - Epigravettian	Phalanx 1 (II-V)	32.2		12.1	7.8	10.1									
**R41**	Reworked - Epigravettian	Phalanx 1 (II-V)	29.3		12.6	7.4	10									
**R57**	Reworked - Epigravettian	Phalanx 1 (II-V)			11											
**R7**	Layers 6-10?	Phalanx 2 (II-V)	18		10	8	10									
**R46**	Reworked - Epigravettian	Phalanx 2 (III-IV)			10.2											
**R67**	Reworked - Epigravettian	Phalanx 2 (III-IV)			9.4											

Supplementary Table 8 | Final Epigravettian wolf measurements from Grotta Romanelli.

Grotta Romanelli			Measurements								
**N°**	**Layer**	**Element**	**GL**	**GB**	**Bp**	**SD**	**Bd**	**SDO**	**DPA**	**LAR**	**DC**
**2720**	Terre brune	Ulna						20.2	24		
**5793**	Terre brune	Mc I	24.9				6.5				
**6452**	Terre brune	Mc II	73.4				11.9				
**6454**	Terre brune	Mc IV	82.7				11.1				
**6456**	Terre brune	Mc IV	79.9				11.7				
**R 40 II**	Terre brune	Mc V					10.6				
**2721**	Terre brune	Pelvis								25.4	
**6447**	Terre brune	Femur			50.4						24.4
**6451**	Terre brune	Calcaneum	55.8	22.7							
**5810**	Terre brune	Mt III	91.3				12.3				
**R 40 I**	Terre brune	Phalanx I (II-V)	30.5		10.9	6.6	9.5				
**R 58 VI**	Terre brune	Phalanx I (II-V)	29.1		10.7	7.2	8.9				
**R 53 tb**	Terre brune	Phalanx I (II-V)	29.5			7.2					
**5807**	Terre brune	Phalanx I (III-IV)	34.7		11	7.1	9.9				
**R tb**	Terre brune	Phalanx II (III-IV)	23.3		10.3	6.8	10				
**5808**	Terre brune	Phalanx II (III-IV)	25		10	6.4	9.5				
**5795**	Terre brune	Phalanx II (III-IV)	21.1		10.1	6.9	9.9				
**P6265**	Terre brune	M/1	30.4	13							
**P6445**	Terre brune	M/1	25.7	10							
**3596_3**	Terre brune	M/1	24.7	9.1							
**P3591**	Terre brune	M/1	23.7	8.8							

Supplementary Table 9 | List of samples analysed by µCT and related acquisition parameters.

DSS: distance source-sample; DSD: distance source-detector.

Specimen	DSS (mm)	DSD (mm)	Voxel size (µm)	Energy (kV)	Current (µA)	Exp time (s)	Filter	Projections
**Dog and wolf first metacarpals**								
13247, 17165	155	445	17,42	110	90	0,5*4	2,5 mm Al	2400
52, 196, 1971	155	445	17,42	110	90	0,5*4	2,5 mm Al	2400
214, 180	155	445	17,42	110	90	0,5*4	2,5 mm Al	2400
353, 361	155	415	18,67	80	100	4*05	2 mm Al	2400
R23, R24	155	445	17,42	110	90	1.2	3 mm Al	2400
C Lj, L Lj	155	455	17,03	100	100	1	2 mm Al	2400
139, 138	175	425	20,59	110	90	0,8	1 mm Al	1440
362	155	555	13,96	80	120	1	2 mm Al	2400
**Dog first phalanx**								
21875	155	555	13,96	80	120	1	2 mm Al	2400
**Dog and wolf lower first molars**								
877	155	455	17,03	100	100	1*4	2 mm Al	2400
358	155	455	17,03	110	90	1	2 mm Al	2400
377	325	805	20,19	110	90	2	1 mm Al	1440
196	325	805	20,19	110	90	2,5	1 mm Al	1440
376	325	805	20,19	110	90	2,5	1 mm Al	1440
Vpa6381	325	805	20,19	110	90	2,5	1 mm Al	1440
M1_Sc1	325	805	20,19	110	90	2,5	1 mm Al	1440
Vpa6312	325	805	20,19	110	90	2,5	1 mm Al	1440
375	325	805	20,19	110	90	2,5	1 mm Al	1440
52	325	805	20,19	110	90	2,5	1 mm Al	1440
378	325	805	20,19	110	90	2,5	1 mm Al	1440
R38	325	805	20,19	110	90	2,5	1 mm Al	1440
353	325	805	20,19	110	90	2,5	1 mm Al	1440

359	325	805	20,19	110	90	2,5	1 mm Al	1440
TS6	325	805	20,19	120	80	2,5	2 mm Al	1440
TS9	325	805	20,19	110	90	2,5	2 mm Al	1440
TS10	325	805	20,19	110	90	2,5	2 mm Al	1440
TS11	325	805	20,19	110	90	2,5	2 mm Al	1440
TS13	325	805	20,19	110	90	2,5	2 mm Al	1440
TS8	325	805	20,19	110	90	2,5	2 mm Al	1440
TS3	325	805	20,19	110	90	2,5	2 mm Al	1440
TSnonum	325	805	20,19	110	90	2,5	2 mm Al	1440
Vpa6304	325	805	20,19	110	90	2,5	2 mm Al	1440
Vpa6309	325	805	20,19	110	90	2,5	2 mm Al	1440
SC3	325	805	20,19	110	90	2,5	2 mm Al	1440
Vpa6302	325	805	20,19	110	90	2,5	2 mm Al	1440
Vpa6303	325	805	20,19	110	90	3	2 mm Al	1440
Vpa6296	325	805	20,19	110	90	3	2 mm Al	1440
TS7	325	805	20,19	110	90	2,5	2 mm Al	1440
M766	325	805	20,19	110	90	2,5	2 mm Al	1440
551	325	805	20,19	110	90	2,5	2 mm Al	1440
757	325	805	20,19	110	90	2,5	2 mm Al	1440
Chiostraccio	175	425	20,59	110	90	0,8	1 mm Al	1440
70	175	425	20,59	110	90	0,8	1 mm Al	1440
1359	175	425	20,59	110	90	0,8	1 mm Al	1440
1	175	425	20,59	110	90	0,8	1 mm Al	1440
2	175	425	20,59	110	90	0,8	1 mm Al	1440
95F	175	425	20,59	110	90	0,8	1 mm Al	1440
fis_135	175	425	20,59	110	90	0,8	1 mm Al	1440
fis_136	175	425	20,59	110	90	0,8	1 mm Al	1440
fis_139	175	425	20,59	110	90	0,8	1 mm Al	1440
3596_3	175	425	20,59	110	90	0,8	1 mm Al	1440
P6450	175	425	20,59	110	90	0,8	1 mm Al	1440
P6445	175	425	20,59	110	90	0,8	1 mm Al	1440
P6265	175	425	20,59	110	90	1	1 mm Al	1440

Supplementary Table 10 | Specimens used for geometric morphometric analyses.

**Taxonomy and chronology**	**N°**	**Group for shape analysis**
Upper Palaeolithic canid – Grotta Paglicci	R4	projected a posteriori
Upper Palaeolithic canid – Grotta Romanelli	P6450	projected a posteriori
Dog – present-day	1	Dog
Dog – present-day	2	Dog
Dog – present-day	70	Dog
Dog – present-day	196	Dog
Dog – present-day	757	Dog
Dog – present-day	1359	Dog
Dog – present-day	95F	Dog
Dog – present-day	Chiostraccio	Dog
Dog – present-day	M766	Dog
Dog – present-day	TS nonum	Dog
Dog – present-day	TS10	Dog
Dog – present-day	TS11	Dog
Dog – present-day	TS12	Dog
Dog – present-day	TS3	Dog
Dog – present-day	TS6	Dog
Dog – present-day	TS7	Dog
Dog – present-day	TS8	Dog
Dog – present-day	TS9	Dog
Dog - archaeological – Holocene – NHMT	SC1	Dog
Dog - archaeological – Holocene – NHMT	SC3	Dog

Dog - archaeological – Holocene – NHMT	Vpa6831	Dog
Wolf - present-day (zoo) – University of Siena	52	Wolf
Wolf - present-day (zoo) – University of Siena	180	Wolf
Wolf - present-day (wild) – University of Siena	353	Wolf
Wolf - present-day (wild) – University of Siena	357	Wolf
Wolf - present-day (wild) – University of Siena	358	Wolf
Wolf - present-day (wild) – University of Siena	359	Wolf
Wolf - present-day (wild) – University of Siena	375	Wolf
Wolf - present-day (wild) – University of Siena	376	Wolf
Wolf - present-day (wild) – University of Siena	377	Wolf
Wolf - present-day (wild) – University of Siena	378	Wolf
Wolf - present-day (wild) – NHMT	551	Wolf
Wolf - present-day (wild) – Fisiocritici Academy	fis_135	Wolf
Wolf - archaeological - Late Pleistocene – NHMT	Vpa0090	Wolf
Wolf - archaeological - Holocene – NHMT	Vpa6312	Wolf
Wolf - archaeological - Holocene – NHMT	Vpa6296	Wolf
Wolf - archaeological – Holocene – NHMT	Vpa6302	Wolf
Wolf - archaeological - Holocene – NHMT	Vpa6303	Wolf
Wolf - archaeological - Holocene – NHMT	Vpa6309	Wolf
Wolf - archaeological - Middle Palaeolithic	3596_3	Wolf
Wolf - archaeological - Upper Palaeolithic – Grotta Romanelli	P6265	Wolf
Wolf - archaeological - Upper Palaeolithic – Grotta Romanelli	P6445	Wolf.
Wolf - archaeological - Upper Palaeolithic – Grotta Paglicci	R38	Wolf

Wolf - archaeological - Upper Palaeolithic – Grotta Paglicci	17775	Wolf

Supplementary Table 11 | Primers used in this study.

Amplicon size (bp)	Primer name	Primer range	Primer sequence 5’-3’
8,528	F1	F2360-F2379	CCCTAGGGATAACAGCGCAA
	R1	R10869-10888	CTACGGCCTCCTTCAACCAA
7,641	F2	F10532-10552	CAGCCTCTGAACTCATCTTCT
	R2	R2872-R2891	CCCTATTGCCGGCTCCATAG

Data S1 (separate file).

Skeletal measurements of wolves and dogs compared to the standard.

Data S2 (separate file).

Measurements of modern Italian wolves.

Data S3 (separate file).

Acquisition data of MicroCt scans.

Data S4 (separate file).

aDNA. Accession numbers of comparative sample.
